# Ca’ Granda, *Hortus simplicium*: Restoring an Ancient Medicinal Garden of XV–XIX Century in Milan (Italy)

**DOI:** 10.3390/molecules26226933

**Published:** 2021-11-17

**Authors:** Martina Bottoni, Fabrizia Milani, Paolo M. Galimberti, Lucia Vignati, Patrizia Luise Romanini, Luca Lavezzo, Livia Martinetti, Claudia Giuliani, Gelsomina Fico

**Affiliations:** 1 Department of Pharmaceutical Sciences, University of Milan, Via Mangiagalli 25, 20133 Milan, Italy; martina.bottoni@unimi.it (M.B.); fabrizia.milani@unimi.it (F.M.); patrizialuise.romanini@studenti.unimi.it (P.L.R.); luca.lavezzo@studenti.unimi.it (L.L.); gelsomina.fico@unimi.it (G.F.); 2 Ghirardi Botanic Garden, Department of Pharmaceutical Sciences, University of Milan, Via Religione 25, 25088 Toscolano Maderno, Italy; 3 Fondazione IRCCS Ca’ Granda Ospedale Maggiore Policlinico, Via Francesco Sforza 28, 20122 Milan, Italy; archivio@policlinico.mi.it; 4 Landscape Ecomuseum of Parabiago, P.za della Vittoria 7, 20015 Milan, Italy; lucia.vignati@comune.parabiago.mi.it; 5 Department of Agricultural and Environmental Sciences-Production, Landscape, Agroenergy, University of Milan, Via Celoria 2, 20133 Milan, Italy; livia.martinetti@unimi.it

**Keywords:** *Hortus simplicium*, ethnobotany, ethnopharmacology, medicinal plants, botanic garden, restoration

## Abstract

This work is based on the study of 150 majolica vases dated back to the mid XVII century that once preserved medicinal remedies prepared in the ancient Pharmacy annexed to the Ospedale Maggiore Ca’ Granda in Milan (Lombardy, Italy). The *Hortus simplicium* was created in 1641 as a source of plant-based ingredients for those remedies. The main objective of the present work is to lay the knowledge base for the restoration of the ancient Garden for educational and informative purposes. Therefore, the following complementary phases were carried out: *(i)* the analysis of the inscriptions on the jars, along with the survey on historical medical texts, allowing for the positive identification of the plant ingredients of the remedies and their ancient use as medicines; *(ii)* the bibliographic research in modern pharmacological literature in order to validate or refute the historical uses; *(iii)* the realization of the checklist of plants potentially present in cultivation at the ancient Garden, concurrently with the comparison with the results of a previous in situ archaeobotanical study concerning pollen grains. For the species selection, considerations were made also regarding drug amounts in the remedies and pedoclimatic conditions of the study area. Out of the 150 vases, 108 contained plant-based remedies, corresponding to 148 *taxa*. The remedies mainly treated gastrointestinal and respiratory disorders. At least one of the medicinal uses was validated in scientific literature for 112 out of the 148 examined species. Finally, a checklist of 40 *taxa*, presumably hosted in the *Hortus simplicium*, was assembled.

## 1. Introduction

Ospedale Maggiore Ca’ Granda (Milan, Lombardy, Italy), today known simply as Policlinico, is considered one of the oldest hospitals in all of Italy. Founded in 1456 at the behest of Francesco Sforza, Duke of Milan, and based on a design by the architect Antonio Averlino, its main purpose was to provide free medical care to the poorest inhabitants of the city as well as improve the efficiency of the healthcare system across the diocese territory. For centuries, this institution was considered a model in the construction of many other European hospitals [1]. The building included also a Pharmacy, place of research, preparation, and distribution of different remedies, as testified by a historical document from 1470 [2,3,4,5]. Between 1640 and 1643, the Hospital commissioned ceramist Michele Valli, from Lodi, to manufacture 575 majolica pots for the ingredients of the annexed Pharmacy, while new supplies of vases were secured over the course of the first half of the XVIII century [6]. At the dawn of World War II, 196 pots were still viable. However, after the bombings, only 150 remained unscathed to this day. Of these, 37 were part of the original production, while the remaining ones were realised during the 1700s. The aforementioned collection is currently preserved by the Service for Cultural Assets of the Policlinico [7]. The vases were used to preserve both single ingredients and complex remedies. These specimens were often plant-based and were processed in the Pharmacy to be administered to the patients of the Hospital. In 1641, a Garden of Simples, annexed to the Hospital, was built. Gardens of Simples, also known as *Horti simplicium*, are considered to be precursors of the modern Botanic Gardens. Originally, back in the Middle Ages, Gardens of Simples were structures connected to ancient monasteries for the cultivation and study of medicinal plants. Later on, they became more prominent in universities, where the plants were used primarily for educational purposes. In contrast, the Garden of the Ospedale Maggiore Ca’ Granda of Milan was annexed to a hospital and the plants therein ended up being directly part of the remedies concocted for the patients [4]. With the emergence of pharmaceutical chemistry in the first half of the XIX century, the ancient Garden gradually lost its relevance as a source of medicinal plants and began to be used as an ornamental green area, no longer intended for the cultivation of medicinal species. During the 1930s, the layout of the Garden was modified due to renovation works of the main building. In the 1960s, additional construction works determined a gradual reduction of the Garden surface area. Today, the area where the ancient Garden stands covers only 680 m^2^, 520 of which are simple lawns [4].

The work presented herein aims at laying the scientific knowledge basis for the future restoration of the ancient Garden of Simples, in the framework of the historical value and educational enhancement of a little-known cultural heritage in Milan. A multidisciplinary approach of investigation was adopted, beginning from the study of the actually preserved 150 majolica jars. The work included complementary subsequent phases: (1) historical survey, with the purpose of defining the composition of the remedies contained in the jars and their historical medical use, focusing on plant-based ingredients; (2) pharmacological research, performed through the consultation of the current relevant scientific literature, in order to either validate or refute the ancient medicinal uses of the plants surveyed; (3) compilation of a checklist of *taxa* that were potentially present in cultivation in the ancient Garden of Simples.

## 2. Results and Discussion

### 2.1. Inscriptions Analysis and Interpretation

**Figure 1:**
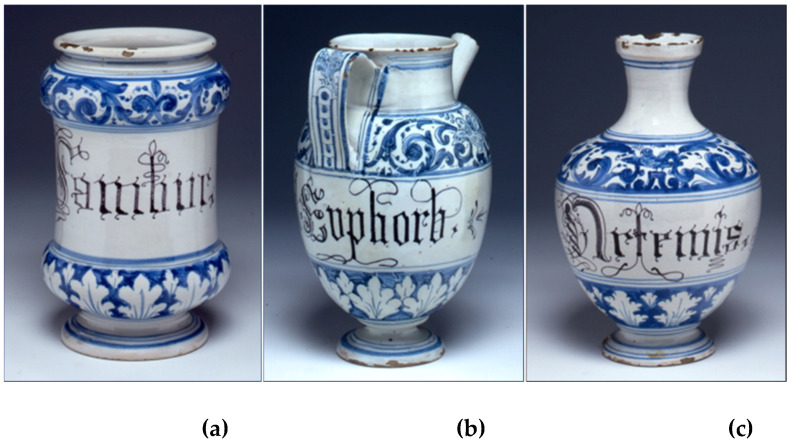
Majolica vases: (**a**) spool *albarello*; (**b**) *orciolo*; (**c**) spheroidal bottle. (Images owned by *Fondazione IRCCS Ca’ Granda Ospedale Maggiore Policlinico*, Milan.)

The 150 jars were categorised into three types (Figure 1): spool *albarelli* (*albarelli a rocchetto*, or slender terracotta containers with a short neck and a large opening) of two different sizes (Figure 1a); jugs (*orcioli*, or pot-bellied containers with a hole at the bottom that allows easy spilling of the contents, Figure 1b); and spheroidal bottles (Figure 1c). The pots are made of white majolica with bear blue decorations and the inscriptions are in old Gothic style and black ink. Each vase is numbered progressively. The inscriptions are written in Latin or vulgar Italian and are for the most part abbreviated, thus making information concerning the ingredients and the types of preparation sometimes hard to discern.

In some cases, the abbreviations were hard to interpret also due to potential spelling mistakes made by the decorator, or to the presence of uncommon and unfamiliar words. As a way of example, we cite vase n. 33, *Syrupus d. Duab. Rad*., which was the extremely contracted version of the Latin *Syrupus de Duabus Radicibus*. For these reasons, the inscriptions needed attentive reading. Based on the interpretation of the labels and the origin of the main ingredients, the jars were further categorised as plant-based (108 vases; vase n. 17, which contained as the main ingredient mushrooms belonging to the genus *Agaricus,* was also included in this category), animal-based (13), mineral-based (8), or unknown origin (21; in these cases, both the deciphering of the inscriptions and the historical survey yielded no usable results). Finally, the plant-based category was subdivided into 15 groups, based on the type of preparation: *aqua* (27 vases; aqueous extract); *syrupus* (21; syrup); *trochiscus* (18; dosage form similar to granules); *oleum* (12; oleolytes); *unguentum* (9; ointment); *electuarium* (6; electuary); *pilulae* (5; dosage form similar to tablets); *mel. ros* (2; honey-based composition); *pulvis* (2; powder); *reb/roab* (2; condensed syrup); *oxymel* (2; liquid preparation based on honey and vinegar); *diatrum* (1; preparation made up of three components); *emplastrum* (1; poultice); *floris* (1; flowers); *opiatus poter.* (1; opium-based preparation). It is worth recalling that the remaining jars represent only a limited part of all the products that could originally be found in the Pharmacy, thus giving us only partial knowledge concerning the remedies used. See Table 1 for details.

### 2.2. Plant Species in the Remedies and Validation of the Historical Medicinal Use

A total 108 plant-based remedies were cross-referenced on a wide type of historical sources, such as ancient pharmacopoeias, medical texts, and almanacs published between the XV and the XIX century [8,9,10,11,12,13,14,15,16,17,18,19,20,21,22,23,24,25,26,27,28,29,30,31,32,33,34,35,36,37,38,39,40,41,42,43,44,45,46,47,48,49,50,51,52,53,54,55,56,57,58,59,60,61,62,63,64,65,66,67,68,69,70,71,72,73,74,75,76,77,78]. In this manner, a total of 148 plant *taxa*, belonging to 58 different botanical families, were found. The complete list is available in Table S1. The most cited families were Apiaceae and Lamiaceae (16 *taxa*; 10.8%), Compositae (12; 8.1%), Rosaceae (9; 6.1%), and Leguminosae (7; 4.7%), while the most represented genera were *Mentha*, *Origanum*, and *Prunus* (3 species each) followed by *Commiphora* (2), *Ferula* (2), and *Pistacia* (2).

The historical medicinal uses documented for the 108 plant-based remedies concern the treatment of the following ailments: digestive tract disorders (diarrhoea, constipation, gastritis and ulcers, intestinal parasites; 46 vases); general condition (anti-inflammatory, antipyretics, etc.; 30); respiratory tract infections (cough, mucus, tuberculosis, etc.; 28); nervous system disorders (tonics, relaxants, stimulants, etc.; 23); skin diseases and traumas (scabies and other skin parasites, irritations, wounds, etc.; 21); circulatory/lymphatic system disorders (microcirculation, haemorrhages, spleen inflammation, etc.; 21); gynaecological disorders, obstetric, and puerperal problems (emmenagogue activity, facilitate birth, etc.; 18); urinary tract disorders (kidney stones, diuretics, etc.; 14); musculoskeletal system disorders and traumas (muscle and/or joint pain, arthritis, arthrosis, gout, etc.; 12); ‘others’ (venereal diseases, other pathologies, leftover from the official medicine of the time; 10); oropharyngeal cavity affections (gingivitis, other inflammations, etc.; 2); afflictions of the ear (otitis, etc.; 2); ophthalmic ailments (inflammations, eye care, etc.; 2). Regarding the plant species used to treat the different pathologies, 83 were ingredients in remedies for digestive tract disorders, 76 for circulatory/lymphatic system disorders, and 69 for respiratory tract infections. See Table 2 for complete details.

The survey of the modern pharmacological literature highlighted that at least one historical therapeutic effect was validated for 112 *taxa* out of 148. On the contrary, it is noteworthy that the effects reported in literature for *Matricaria chamomilla* L. were opposite in comparison with the uses documented by the historical sources; specifically, in the past it was used in a laxative remedy [20], while the modern literature referred to antidiarrheal properties [79]. Of the consulted literature contributions, 17 reported different plant parts when compared with the ones used in the past. For example, historical sources cited roots and seeds to be used for *Asarum europaeum* L. [9,13,19], while the current pharmacological studies were focused on the plant aerial parts [80].

For each of the *taxa* found in plant-based remedies, the exhaustive comparison with modern pharmacological literature data was presented hereafter (for the complete dataset, see Table S1): *Acacia senegal* (L.) Willd., anti-inflammatory [81]; *Achillea millefolium* L., antibacterial, antiulcer, emmenagogue [82]; *Acorus calamus* L., antiasthmatic, antidiarrheal, anti-inflammatory, kidney stones, diuretic, hypocholesterolemic [83,84,85,86]; *Adiantum capillus-veneris* L., antimicrobial, anti-inflammatory, antipyretic, antiviral [87,88,89,90]; *Agaricus bisporus* (J.E. Lange) Imbach, antibacterial, antiviral, anti-inflammatory, gastritis, stomach disorders, immunomodulant [91,92]; *Agrimonia eupatoria* L., anti-inflammatory (aerial parts), antioxidant (aerial parts) [93]; *Aloe* spp., antibacterial, wound healing, airways prophylaxis, laxative, vermifuge [94,95,96,97,98]; *Althaea officinalis* L., anti-inflammatory, expectorant [99,100]; *Anchusa officinalis* L., antidiabetic, anti-inflammatory, antioxidant [101]; *Anethum graveolens* L., antimicrobial, anti-inflammatory, analgesic [102]; *Angelica* spp., antibacterial, anti-inflammatory, antioxidant, antipyretic, bronchodilator (epigeal part) [103]; *Artemisia absinthium* L., antibacterial, anthelmintic, antifungal, antiprotozoal, antiviral, antioxidant, anti-inflammatory, antipyretic, analgesic, antiulcer, digestive, immunomodulant, kidneys, wounds, jaundice, neuroprotector [104]; *Artemisia* spp., hepatoprotector (leaves) [105]; *Asarum europaeum* L., antialzheimer, antitumoral (aerial parts) [80,106]; *Asparagus officinalis* L., laxative [107]; *Asplenium scoloprendrium* L., antioxidant [108]; *Athamanta turbith* (L) Brot., antimicrobial [109]; *Borago officinalis* L., antiasthmatic (leaves), anti-inflammatory (seeds), antioxidant, spasmolytic (leaves), circulation (leaves) [110,111,112,113]; *Boswellia serrata* Roxb. ex Colebr., antibacterial, antifungal, anti-inflammatory, antioxidant, antimicrobial, asthma, skin wounds, sedative [114,115,116]; *Bryonia* spp., anti-inflammatory [117]; *Carum carvi* L., diuretic, dyspepsia, emmenagogue, gastrointestinal disorders [118,119]; *Capparis spinosa* L., antimicrobial, anti-inflammatory, hepatoprotector (aerial parts), hypocholesterolemic (fruit), hypoglycaemic (fruit), hypolipidemic (fruit) [120,121,122]; *Centaurium erythraea* Rafn., antipyretic, hepatoprotector [123,124]; *Centaurea benedicta* (L.) L., antischistosomiasis, wounds, antiulcer [125,126]; *Ceterach officinarum* Willd., antioxidant [127,128]; *Cichorium endivia* L., antioxidant (hypogeal parts), hepatoprotector [129,130]; *Citrus medica* L., antibacterial [131]; *Cinnamomum camphora* (L.) J. Presl, antifungal, anti-inflammatory, antiparasitic [132,133,134]; *Cinnamomum verum* J. Presl, airways, gastrointestinal disorders, strengthens the nervous system [135,136,137]; *Citrullus colocynthis* (L.) Schrad., anti-inflammatory [138]; *Commiphora gileadensis* (L.) C. Chr., antibacterial [139]; *Commiphora myrrha* (Nees) Engl., antibacterial, antifungal, anti-inflammatory, antioxidant, antiseptic, antihistamine, digestive, emmenagogue, stimulates the urinary tract, gingivitis, airways [140,141,142]; *Crocus* spp., gastrointestinal disorders, airways, sedative [143,144,145,146]; *Cucumis melo* L., anti-inflammatory [147]; *Cuminum cynumum* L., emmenagogue, gastrointestinal disorders [118]; *Curcuma longa* L., gastrointestinal disorders, airways [148,149]; *Cyclamen hederifolium* Aiton, anti-inflammatory [150,151]; *Cynoglossum officinale* L., analgesic, antibacterial, antihemorrhagic, anti-inflammatory, antiseptic [152]; *Cyperus esculentus* L., anti-inflammatory [153]; *Daucus carota* L., circulation [154]; *Dorema ammoniacum* D. Don, analgesic, antibacterial, anti-inflammatory, antiseptic, antiviral, kidney stones, depurative, dermatitis, diuretic, laxative, neuroprotector, airways [155,156,157,158]; *Drimia maritima* (L.) Stearn, antimicrobial, antioxidant, antitumoral, circulation [159]; *Dryopteris filix-mas* (L.) Schott, anti-inflammatory [160]; *Eryngium maritimum* L., antioxidant [161]; *Eupatorium cannabinum* L., anti-inflammatory, choleretic, hepatoprotector [162,163]; *Euphorbia* spp., laxative, vermifuge/anthelmintic [164,165]; *Ferula gummosa* Boiss., antibacterial (E.O. from the seeds), anti-inflammatory [166,167,168]; *Foeniculum vulgare* Mill., antimicrobial, blood depurative [169]; *Fumaria officinalis* L., anti-inflammatory, diuretic [170,171]; *Galega officinalis* L., antibacterial [172]; *Glechoma hederacea* L., anti-inflammatory [173]; *Glycyrrhiza glabra* L., antibacterial, anti-inflammatory, antiparasitic, antihistamine, airways [174,175,176]; *Hordeum vulgare* L., antidiarrheal, constipation, expectorant (aerial parts, whole fruits) [177,178]; *Hypericum perforatum* L., antibacterial, anti-inflammatory [179,180]; *Larix* spp. (or *Pinus* spp., or *Picea* spp.), antibacterial, anti-inflammatory, antioxidant, airways [181,182,183]; *Lavandula dentata* L. anti-inflammatory, antiasthmatic, antioxidant [184]; *Lilium candidum* L., anti-inflammatory [185]; *Linum usitatissimum* L., anti-inflammatory [186]; *Malus domestica* Borkh., antidiarrheal, anti-inflammatory, airways [187,188,189]; *Matricaria chamomilla* L., antidiarrheal, spasmolytic, antiulcer, gastrointestinal disorders [79,190]; *Melissa officinalis* L., spasmolytic [191]; *Mentha pulegium* L., antimicrobial, circulation [192,193]; *Myristica fragrans* Houtt., analgesic, antibacterial, anticonvulsant, anti-inflammatory, antioxidant, stomach-ache [194,195,196,197]; *Myrtus communis* L., antioxidant, antiulcer, neuroprotector [198,199,200]; *Narthex asafoetida* Falc. ex Lindl., spasmolytic [201,202]; *Nigella damscena* L., antioxidant, diuretic [203]; *Nigella sativa* L., anti-inflammatory, antioxidant, diuretic, emmenagogue [203,204]; *Olea europaea* L., antibacterial (post press waste water), anti-inflammatory [111,205,206,207]; *Origanum majorana* L., strengthens the nervous system [208]; *Papaver somniferum* L., analgesic, antidiarrheal, excitant, neuroprotector, airways [209,210,211,212,213]; *Petasites hybridus* (L.) G. Gaertn., B. Mey. and Scherb., antiulcer, expectorant [214]; *Petroselinum crispum* (Mill.) Fuss, antioxidant, diuretic [215]; *Pimpinella anisum* L., antimicrobial, antioxidant, airways, gastrointestinal disorders [216]; *Pimpinella saxifraga* L., antibacterial [217]; *Piper longum* L., anti-inflammatory, antioxidant [218]; *Piper nigrum* L., anti-inflammatory, antioxidant, antiparasitic, digestive [219,220,221]; *Pistacia lentiscus* L., kidney stones (fruit), anti-inflammatory, antioxidant, digestive, hypoglycaemic [222,223]; *Pistacia terebinthus* L., antioxidant, antimicrobial, antiviral [222]; *Polypodium vulgare* L., analgesic, antibacterial, antiviral, digestive, laxative, scurvy [224]; *Portulaca oleracea* L., antihypoxia, anti-inflammatory, antioxidant, hepatoprotector, neuroprotector [225]; *Potentilla erecta* (L.) Raeusch., antibacterial [226]; *Prunus domestica* L., antihistamine [227]; *Prunus dulcis* (Miller) D.A. Webb, anti-inflammatory, emollient (leaves) [228,229,230]; *Prunus persica* (L.) Batsch, laxative [231]; *Pterocarpus santalinus* L.f., antibacterial, anti-inflammatory [232,233]; *Pulmonaria officinalis* L., antioxidant [234]; *Ruscus aculeatus* L., spasmolytic (aerial parts) [235]; *Rheum officinale* L., anti-inflammatory, antioxidant, gastrointestinal disorders, airways, thermogenic [236,237]; *Rosa* spp., antibacterial, anti-inflammatory, laxative, antiviral [238,239,240,241,242]; *Rosmarinus officinalis* L., antimicrobial, anti-inflammatory [243,244]; *Rubia tinctorum* L., gastrointestinal disorders [245]; *Rubus ulmifolius* Schott, anti-inflammatory [246]; *Rumex conglomeratus* Murray, antibacterial, antioxidant [247,248]; *Ruta graveolens* L., antibacterial, anti-inflammatory, antipyretic [249,250]; *Sambucus ebulus* L., antibacterial, anti-inflammatory, antioxidant, diuretic, soothing [251,252,253,254,255,256,257]; *Sambucus nigra* L., diaphoretic, airways viral infections, soothing [251,258,259,260,261]; *Sanguisorba officinalis* L., circulation [262]; *Santalum album* L., antibacterial, anti-inflammatory [263,264]; *Saponaria officinalis* L., antiviral [265]; *Scorzonera* spp., antibacterial, antimicrobial, antifungal, anti-inflammatory, antinematodes, wounds [266,267,268]; *Symphytum officinale* L., anti-inflammatory [269]; *Tanacetum parthenium* (L.) Shc.bip., analgesic, anti-inflammatory, spasmolytic [270,271]; *Teucrium scordium* L., gastrointestinal disorders [272]; *Valeriana* spp., gastrointestinal disorders [273]; *Thymus* spp., anticonvulsant, skin diseases [274,275]; *Trigonella foenum-graecum* L., anti-inflammatory [276]; *Triticum aestivum* L., antioxidant, cardio protector (leaves) [277]; *Veronica* spp., anti-inflammatory [278]; *Viola tricolor* L., anti-inflammatory [279]; *Vitex agnus-castus* L., anti-inflammatory [280]; *Vitis vinifera* L., anti-inflammatory [281]; *Zingiber officinale* Roscoe, gastric acidity, stomach depurative, stomach-ache, vermifuge/anthelmintic [282,283,284].

### 2.3. Plant Species Checklist for the Restoration of the Ancient Garden of Simples

The historical and pharmacological bibliographic research based on the inscriptions of the jars allowed listing of the 148 plant *taxa* that were actively employed at the Ospedale Maggiore Ca’ Granda in Milan. If the ancient Pharmacy was indeed the place of manufacture and distribution of the medicinal remedies, it is well-documented that since 1641, the Garden of Simples was the place of cultivation of the plants that made up the remedies themselves [4]. However, to this day, there is very little information concerning the pool of plants hosted in the Garden. The results of the archaeological and palynological analysis performed by Bosi et al. [4] on both plant remains and pollen grains recovered in the area of study, represent a first attempt to resolve this pivotal issue. As a matter of fact, concerning the herbaceous plants, the authors recovered pollen grains belonging to several species of the Apiaceae (probably including *Carum carvi* L., *Pastinaca sativa* L., *Anethum graveolens* L., *Aethusa cynapium* L., and *Pimpinella anisum* L. Still) and Compositae families (with *Calendula officinalis* L., *Centaurea benedicta* (L.) L., and maybe *Centaurea jacea* L.). Additionally, further *taxa* belonging to different families were identified, such as species of the genera *Hypericum*, *Euphorbia*, *Mercurialis*, *Mentha*, *Allium*, and *Reseda*. The remaining pollen residues turned out to be more difficult to interpret, because it could belong to species hosted in the Garden, to plants cultivated nearby, or even weeds. These included *Papaver* spp., *Potentilla* spp., *Artemisia* spp., and *Brassica* spp. The woody species, on the other hand, presented a more difficult challenge. According to Bosi’s team [4], these plants could have been cultivated both for medicinal purposes and as ornamentals. Among these, the following species were identified: evergreen species belonging to the genera *Buxus* and *Juniperus*, and fruit-bearing trees such as *Morus nigra* L., *Cydonia oblonga* Mill., *Juglans regia* L., *Prunus* spp. (perhaps *P. avium* L.), and *Vitis* spp. (most likely *V. vinifera* L.). Additional species that were present at the time were *Humulus lupulus* L., *Fraxinus ornus* L., *Cornus mas* L., *Olea europaea* L., and *Castanea sativa* L. Nevertheless, according to the authors, it is unreasonable to completely exclude the possibility that these plant remains did not come to be at the Garden from neighbouring areas of Lombardy’s territory.

It should also be considered that of the 148 *taxa*, 76% are autochthonous, growing spontaneously across Italy. However, Milan’s pedoclimatic conditions of the time must also be taken into account. Some species could not have been cultivated in the Garden back in the XVII century due to their thermal requirements, regardless of their presence in the rest of the country. Among these were *Cinnamomum camphora* (L.) J. Presl, *Cinnamomum verum* J. Presl, *Convolvulus scammonia* L., *Curcuma longa* L., *Myristica fragrans* Houtt., and *Pistacia terebinthus* L. On the other hand, other species could have been cultivated in the Garden by taking special precautions, most likely protecting them from cold weather. Examples of these plants are *Capparis spinosa* L., *Drimia maritima* (L.) Stern, *Glycyrrhiza glabra* L., and *Myrtus communis* L. Conversely, plant species such as *Citrus limon* L. or *Citrus medica* L., which prefer more temperate climates, could have been hosted in a sunny and sheltered spot. Another important factor to be considered is the limited area dedicated to cultivation (about 680 m^2^). It is, in fact, improbable that there were a great number of arboreal species, as well as *Loranthus europaeus* Jacq., which grows as an epiphyte on trees.

An additional consideration arises on the plant part and the drug amounts used in the remedies production. Hypogeal organs were historically requested for some of the woody species, such as *Capparis spinosa* L. and *Glycyrrhiza glabra* L. Thus, it is reasonable to assume it very unlikely that even a portion of the already limited growing area was dedicated to plants that would have been completely eradicated to obtain the drug. However, these species could be considered excellent additions for the future restored Garden, for display and educational purposes. Some species were seldomly employed in remedies and/or in very limited amounts (i.e., *Sanguisorba officinalis* L. and *Pulmonaria* spp.). Others, instead, were used in a variety of recipes (i.e., *Artemisia absinthium* L. and *Ruta graveolens* L.). Therefore, it is possible to deduce that the formers were actually grown in the Garden, whereas the latter were most likely supplied from elsewhere. Some historical documents preserved in the Hospital archive were consulted as well (ingredients indexes and supply lists from 1711, 1729, 1760, and 1793 and pharmacopoeias from 1809, 1810–1820, 1819, and 1839 [285,286,287,288,289,290,291,292,293]). Out of the 148 in our complete list, 85 species were cited in the aforementioned documents, 36 of which were in at least 5 of them. It can be thus hypothesised an actual and continuative use of these plants inside the Hospital during the XVIII and XIX centuries. However, some of the supply lists confirm that several of these species and their derivatives were purchased from outside the Pharmacy; these included *Acacia senegal* (L.) Willd. (arabic gum), *Aloe* spp., *Cinnamomum camphora* (L.) J. Presl, *Cinnamomum verum* J. Presl, *Dorema ammoniacum* D. Don (gum ammoniac), *Drimia maritima* (L.) Stern, *Fraxinus ornus* L. (manna), *Glycyrrhiza glabra* L., *Papaver somniferum* L. (opium), *Rheum officinale* Baill., *Rosa* spp. (dried rose petals), and *Tamarindus indica* L. [288]. Other species, cited in at least 5 of the documents but absent from the supply list, could have been hosted in the Garden; among these are *Juniperus communis* L., *Laurus nobilis* L., and *Matricaria chamomilla* L. Finally, plant-based ingredients such as wine and olive oil were used as a base for most of the remedies produced at the Pharmacy. For this reason, it is more than likely that they too were purchased goods, as opposed to being obtained from the cultivation of *Vitis vinifera* L. and *Olea europaea* L. in the Garden. Both these plants would also benefit a potential restored Garden, both as ornamentals and examples of their ancient use. Taking into account all the aforementioned considerations, a list of 40 *taxa*, belonging to 20 botanical families, was compiled. The most represented families are Lamiaceae (12 species) followed by Compositae and Apiaceae (4 species each), while the most represented genera are *Origanum* (3) and *Mentha* (2). The information obtained, along with the 40 species list, represent the basis for the future project of restoration of the ancient *Hortus simplicium*. The complete list is reported in Table 3.

## 3. Materials and Methods

### 3.1. Historical Research

The historical survey led to the identification of the remedies’ ingredients once contained in the jars. First, a list of the 150 vases and their inscriptions was drafted. These inscriptions were then analysed and interpreted with the aid of pharmacopoeias, medical texts, and almanacs published between the XV and the XIX centuries [8,9,10,11,12,13,14,15,16,17,18,19,20,22,23,24,26,27,28,29,30,31,32,33,34,35,36,37,38,39,40,41,42,43,44,45,46,47,48,49,50,51,52,53,55,56,57,58,59,60,61,62,64,65,66,67,68,70,71,72,75,77,78,285,286,287,288,289,290,291,292,293].

Where possible, consultation of writs in vulgar Italian was preferred. The inscriptions were matched directly with the names of the remedies found in the different historical sources. Subsequently, the jars were catalogued according to the origin of either the remedy or the main ingredient. The categories utilised were “plant-based”, “mineral”, and “animal”. All the plant-based ingredients were then tabulated along with the following information: current scientific name (consulting the specialised website http://www.theplantlist.org/, accessed on 30 September 2021), weight, historical medicinal use, and historical source.

### 3.2. Pharmacological Research

Extensive bibliographic research in the pharmacological field was carried out on the plant species obtained during the historical survey phase in order to either validate or refute their ancient medicinal uses. To that end, it was necessary to interpret the historical medical terminology of the pathologies in a modern setting. During this research, several databases were consulted, such as PubMed, Scopus, Google Scholar, and the bibliographic research online tool known as J.A.N.E. A two-step approach was used during the inquiry. Firstly, either the scientific or the common English name of the species was matched with specific keywords related to the categories of pathology that were obtained from historical sources (i.e., *Acorus calamus*, ‘digestive system’ or ‘gastrointestinal disorders’). Secondly, the plant name was matched with the specific pathology or activity (i.e., *Acorus calamus*, ‘laxative’ or ’anti-inflammatory’). The research was primarily focused on systematic reviews and meta-analysis, whenever possible, without applying any year filters. Alternatively, in vitro and in vivo studies, as well as clinical trials, were consulted. The literature inquiry was extended to the mechanism of action, when known. All data were tabulated along with the following information: family, species (scientific and common name), inscription on the vase and inventory number, part of the plant historically used, historical sources, modern use obtained from the historical medicinal use, mechanism of action, and bibliographic references (for complete data, please see Table S1 [8,9,10,11,12,13,14,15,16,17,18,19,20,21,22,23,24,25,26,27,28,29,30,31,32,33,34,35,36,37,38,39,40,41,42,43,44,45,46,47,48,49,50,51,52,53,54,55,56,57,58,59,60,61,62,63,64,65,66,67,68,69,70,71,72,73,74,75,76,77,78,79,80,81,82,83,84,85,86,87,88,89,90,91,92,93,94,95,96,97,98,99,100,101,102,103,104,105,106,107,108,109,110,111,112,113,114,115,116,117,118,119,120,121,122,123,124,125,126,127,128,129,130,131,132,133,134,135,136,137,138,139,140,141,142,143,144,145,146,147,148,149,150,151,152,153,154,155,156,157,158,159,160,161,162,163,164,165,166,167,168,169,170,171,172,173,174,175,176,177,178,179,180,181,182,183,184,185,186,187,188,189,190,191,192,193,194,195,196,197,198,199,200,201,202,203,204,205,206,207,208,209,210,211,212,213,214,215,216,217,218,219,220,221,222,223,224,225,226,227,228,229,230,231,232,233,234,235,236,237,238,239,240,241,242,243,244,245,246,247,248,249,250,251,252,253,254,255,256,257,258,259,260,261,262,263,264,265,266,267,268,269,270,271,272,273,274,275,276,277,278,279,280,281,282,283,284,285,286,294,295,296,297]). 

### 3.3. Checklist of Potentially Cultivated Species at the Ancient Garden of Simples

The list of the plant species obtained from the historical research was compared with data from the archaeobotanical study by Bosi et al. [4]. This study was performed on pollen grains recovered at the area of the ancient Garden. This process allowed for the identification of the plant *taxa* that were potentially present in cultivation at the study area. Each species in the list was then evaluated according to the relative cultivation requirements and the pedoclimatic conditions of the area at the time. Finally, additional considerations were made concerning the part of the plant used in the remedies, the amount in use at the ancient Pharmacy, and XVIII century archived supply ledgers.

## 4. Conclusions

Until the end of the XIX century, official medicine was inextricably linked to the use of plant-based complex remedies. Scientific texts and pharmacopoeias of the time describe elaborate recipes in which animal and mineral ingredients were mixed with plant derivatives in order to produce concoctions that had reportedly almost magical properties. In a time when synthetic drug production was in its infancy and antibiotics did not even exist, in hospitals, doctors administered these peculiar preparations as valid therapies. Ospedale Maggiore Ca’ Granda in Milan, along with the annexed Pharmacy and ancient Garden of Simples, was for centuries the main venue for these ancient therapeutic practices that could be considered both fascinating and debatable. The multidisciplinary approach of research described herein allowed for the coalescence of results obtained from several complementary fields of study, such as history, pharmacology, archaeology, and agronomy, with the common goal of investigating the plant species used in therapy during the XV–XIX centuries. This was made possible thanks to the accurate analysis of the 150 surviving majolica vases actually preserved in the Pharmacy and once used for the conservation of the ingredients and complex remedies administered to the patients of the Hospital. Through this survey, we could speculate on the pool of species that were once hosted in the ancient Garden of Simples. Finally, the information gleamed in this study will prove to be instrumental in the future restoration project of the *Hortus simplicium*, in the framework of the historical value and the educational enhancement of a little-known cultural heritage in Milan.

## Figures and Tables

**Table 1:** List of the 108 plant-based vases, with information about vase number (corresponding to the cataloguing number attributed to each vase in the collection preserved at the Service for Cultural Assets of the Policlinico of Milan), original vase inscription, plant ingredients present in the remedy with the indications of the scientific names, and the historical source.

Vase Number	Vase Inscription	Plant Ingredients (Genus/Species)	Historical Source
2	Aqua Moli	Mythological plant (still unknown) useful for potions and spells	
3	Aqua Aequi.	*Equisetum arvense* L.	[8]
4	Trochiscus Alhandal.	*Citrullus colocynthis* (L.) Schrad.	[9]
6	Unguentum Lapatÿ	*Cinnamomum camphora* (L.) J. Presl, *Rosa* spp., *Rumex conglomeratus* Murray, *Viola* spp.	[10,11]
7	Unguentum Agrippae	*Bryonia* spp., *Drimia maritima* (L.) Stern, *Ecballium elaterium* (L.) A. Rich., *Eryngium maritimum* L., *Pistacia lentiscus* L., *Sambucus ebulus* L.	[10,11]
9	Electuarium Diacurcumae	*Acorus calamus* L., *Artemisia absinthium* L., *Ceterach officinarum* Willd., *Cinnamomum verum* J. Presl, *Commiphora gileadensis* (L.) C. Chr., *Commiphora myrra* Nees, *Crocus* spp. or *Crocus sativus* L., *Curcuma longa* L., *Cyperus esculentus* L., *Daucus carota* L., *Eupatorium cannabinum* L., *Glycyrrhiza glabra* L., *Lavandula dentata* L., *Papaver somniferum* L., *Pimpinella anisum* L., *Rheum officinale* L., *Rubia tinctorum* L., *Teucrium scordium* L., *Valeriana* spp.	[12]
10	Unguentum Rosati	*Prunus dulcis* (Mill.) D.A. Webb, *Rosa* spp.	[10,11]
11	Trochiscus Absÿnthi	*Artemisia absinthium* L., *Asarum europeum* L., *Lavandula dentata* L., *Rosa* spp.	[9]
13	Trochiscus d. Myrtha.	*Myrtus communis* L.	-
14	Conserva Hamech.	*Artemisia absenthium* L., *Citrullus colocynthis* (L.) Schrad., *Pimpinella anisum* L., *Polypodium vulgare* L., *Prunus domestica* L., *Rosa* spp., *Thymus* spp., *Viola* spp.	[9]
15	Unguentum Pectorale	*Althaea officinalis* L., *Anethum graveolens* L., *Myristica fragrans* Houtt., *Prunus dulcis* (Miller) D.A. Webb, *Rosmarinus officinalis* L.	[13]
16	Aqua Aequi.	*Equisetum arvense* L.	[8]
17	Trochiscus de Agarici	*Agaricus campestris* L., *Agaricus bisporus* (J.E. Lange) Imbach, *Zingiber officinale* Roscoe	[10,11,14]
18	Oleum Nucis. mÿrist.	*Myristica fragrans* Houtt.	[15]
19	Electuarium bened. lax.	*Achillea millefolium* L., *Acorus calamus* L., *Alpinia galanga* (L.) Willd., *Apium graveolens* L., *Asparagus officinalis* L., *Athamanta turbith* (L.) Brot., *Carum carvi* L., *Convolvulus scammonia* L., *Crocus* spp. or *Crocus sativus* L., *Dianthus Caryophyllus* L., *Elettaria cardamomum* (L.) Maton, *Euphorbia esula* L., *Foeniculum vulgare* Mill., *Iris tuberosa* L., *Lavandula dentata* L., *Myristica fragrans* Houtt., *Piper longum* L., *Rosa* spp., *Ruscus aculeatus* L., *Saxifraga* spp., *Zingiber officinale* Roscoe	[16]
21	Conserva Boragina.	*Borago officinalis* L.	[17,18]
23	Pilulae Aloe. lota.	*Aloe* spp., *Agaricus bisporus* (J.E. Lange) Imbach or *Agaricus campestris* L., *Rosa* spp.	[10,11,19]
24	Unguentum Artanita	*Aloe* spp., *Capparis spinosa* L., *Citrullus colocynthis* (L.) Schrad., *Commiphora myrrha* (Nees) Engl., *Convolvulus scammonia* L., *Cyclamen hederifolium* Aiton, *Daphne mezereum* L., *Dryopteris filix-mas* (L.) Schott, *Ecballium elaterium* (L.) A. Rich., *Euphorbia* spp., *Ferula persica* Willd., *Iris tuberosa* L., *Lavandula dentata* L., *Matricaria chamomilla* L., *Piper nigrum* L., *Polypodium vulgare* L., *Prunus dulcis* (Mill.) D.A. Webb, *Sambucus ebulus L*., *Tamarix gallica* L., *Vitis vinifera* L., *Zingiber officinale* Roscoe	[20]
26	Oleum Sup. hord.	*Hordeum vulgare* L.	[21]
27	Oxymel Scyll.	*Drimia maritima* (L.) Stearn	[22]
28	Syrupus rosatus solutus cum fumaria	*Fumaria officinalis* L., *Rosa* spp.	[8]
29	Oleum Spica.	*Lavandula dentata* L., *Sesamum indicum* L.	[23]
30	Syrupus d. Pomis.s.	*Malus domestica* (Suckow) Borkh., *Prunus dulcis* (Mill.) D.A. Webb	[9]
31	Oleum Mastyc.	*Pistacia lentiscus* L.	[24]
32	Syrupus de Mÿrtio	*Myrtus communis* L.	[9]
33	Syrupus d. Duab. rad.	*Foeniculum vulgare* Mill., *Petroselinum crispum* (Mill.) Fuss	[25]
34	Oleum Absinthÿ	*Artemisia absinthium* L.	[26]
35	Syrupus heder. terres.	*Glechoma hederacea* L., *Rosa* spp.	[27]
36	Oleum Lil. alb. q.pl.	*Lilium candidum* L.	[28]
41	Electuarium Diaccatol.	*Citrullus colocynthis* (L.) Schrad.	[29]
44	Unguentum Citrini	*Boswellia serrata* Roxb. ex Colebr., *Citrus medica* L., *Cinnamomum camphora* (L.) J. Presl	[30]
45	Trochiscus d. Cappar.	*Acorus calamus* L., *Agrimonia eupatoria* L., *Aristolochia rotunda* L., *Asplenium scolopendrum* L., *Capparis spinosa* L., *Clinopodium nepeta* subsp. *glandulosum* (Req.) Govaerts, *Cyperus esculentus L., Dorema ammoniacum* D. Don, *Nigella damascena* L., *Prunus dulcis* (Miller) D.A. Webb, *Ruta graveolens* L., *Vitex agnus-castus* L.	[9]
46	Pilulae Fetid.	*Narthex asafoetida* Falc. ex Lindl.	[31]
48	Electuarium Diascord.	*Achillea millefolium* L., *Angelica* spp., *Centaurea benedicta* (L.) L., *Galega officinalis* L., *Potentilla erecta* (L.) Raeusch., *Ruta graveolens* L., *Sambucus nigra* L., *Scorzonera* spp., *Teucrium scordium* L.	[32,33]
50	Diatrium. santal.	*Acacia senegal* (L.) Willd., *Astragalus bustillosii* Clos, *Cichorium endivia* L., *Cucumis melo* L., *Glycyrrhiza glabra* L., *Portulaca oleracea* L., *Pterocarpus santalinus* L.f., *Rosa* spp., *Rhaponticum scariosum* Lam., *Santalum album* L., *Viola* spp.	[9]
51	Trochiscus de Mirra	*Artemisia absinthium* L., *Commiphora myrrha (Nees)* Engl., *Cuminum cynimum* L., *Lupinus albus* L., *Rubia tinctorum* L.	[9]
53	Oleum Mitridat. d.	Different species, exact recipe not yet known	
54	Electuarium d. Bac. laur.	*Acorus calamus* L., *Carum carvi* L., *Cuminum cyminum* L., *Laurus nobilis* L., *Nigella sativa* L., *Origanum vulgare* L., *Piper longum* L., *Piper nigrum* L., *Prunus dulcis* (Miller) D.A. Webb, *Ruta graveolens* L.	[9]
55	Opiatus poter.	*Papaver somniferum L*., *Veronica* spp	[34]
57	Trochiscus Aland.	*Citrullus colocynthis* (L.) Schrad.	[9]
60	Extractus Vissi. querc.	*Loranthus europaeus* Jacq.	[35]
61	Unguentum Dialthee sub.	*Althaea officinalis* L., *Larix* spp. or *Pinus* spp. or *Picea* spp. (turpentine), *Larix* spp. or *Pinus* spp. or *Picea* spp. (rosin), *Linum usitatissimum* L., *Trigonella foenum-graecum* L.	[36,37]
62	Reb. Sambuc.	*Sambucus nigra* L.	[38]
64	Conserva Absinth.	*Artemisia absinthium* L.	[39,40]
68	Unguentum Diagridium	*Convolvulus scammonia* L.	Not Found
69	Pilulae de Cinoglo.	*Boswellia serrata* Roxb. ex Colebr., *Commiphora myrrha* (Nees) Engl., *Crocus* spp. or *Crocus sativus* L., *Cynoglossum officinale* L., *Hyoscyamus niger* L., *Papaver somniferum* L., *Viola* spp.	[9]
75	Extractus haed. terrest.	*Glechoma hederacea* L.	[41]
76	Unguentum Lapat.	*Cinnamomum camphora* (L.) J. Presl, *Rosa* spp., *Rumex conglomeratus* Murray, *Viola* spp.	[10,11]
78	Emplastrum crustae panis m.	*Mentha* spp., *Pistacia lentiscus* L., *Pterocarpus santalinus* L.f., *Santalum album* L., *Triticum aestivum* L. subsp. *aestivum*, *Vitis vinifera* L.	[9]
79	Roab. Sambuc.	*Sambucus nigra* L.	[38]
80	Electuarium d. Bac. laur.	*Acorus calamus* L., *Carum carvi* L., *Cuminum cyminum* L., *Laurus nobilis* L., *Nigella sativa* L., *Origanum vulgare* L., *Piper longum* L., *Piper nigrum* L., *Prunus dulcis* (Miller) D.A. Webb, *Ruta graveolens* L.	[9]
81	Pilulae de Amon. q.	*Aloe* spp., *Commiphora myrrha* (Nees) Engl., *Convolvulus scammonia* L., *Dorema ammoniacum* D. Don, *Ferula gummosa* Boiss., *Glycyrrhiza glabra* L., *Pistacia terebinthus* L.	[42]
82	Pulvis hermodac.	*Iris tuberosa* L.	[43]
84	Conserva Rosar.	*Rosa* spp.	[9]
86	Pilulae Masticin.	*Aloe perryi* Baker, *Pistacia lentiscus* L., *Rosa* spp.	[44]
87	Extractus haed. terrest.	*Glechoma hederacea* L.	[41]
92	Syrupus Absynthii	*Artemisia absinthium* L.	[45,46]
93	Mel. ros. sol. com.	*Rosa* spp.	[10,11]
94	Syrupus Cichor. Com. Reub. Gul. -n.	*Cichorium intybus* L., *Rheum officinale* L.	[47,48]
95	Oleum Lil. alb.	*Lilium candidum* L.	[28]
96	Syrupus d. Artemisie q.p.	*Artemisia* spp., *Asarum europaeum* L., *Centaurium erythraea* Rafn, *Cinnamomum verum* J. Presl, *Foeniculum vulgare* Mill., *Juniperus communis* L., *Juniperus sabina* L., *Lavandula dentata* L., *Ligustrum vulgare* L., *Mentha pulegium* L., *Origanum dictamnus* L., *Origanum majorana* L., *Origanum vulgare* L., *Petroselinum crispum* (Mill.) Fuss, *Portulaca oleracea* L., *Ruta graveolens* L.	[13,19]
97	Syrupus Betton.	*Stachys officinalis* (L.) Trevisan	[9]
98	Syrupus Althee. fernet.	*Althaea officinalis* L.	[9]
99	Syrupus d. s. Betton.	*Stachys officinalis* (L.) Trevisan	[9]
100	Syrupus roxato	*Rosa* spp.	[10,11]
101	Oleum Sampsuc.	*Mentha aquatica* L., *Myrtus communis* L., *Olea europaea* L., *Origanum majorana* L., *Thymus* spp.	[49]
102	Oleum de. Rutha.	*Olea europaea* L., *Ruta graveolens* L.	[50]
103	Syrupus cap. Vener.	*Adiantum capillus-veneris* L.	[51]
104	Oleum Hÿperic. q.pl.	*Hypericum perforatum* L., *Olea europaea* L.	[44,52]
105	Syrupus rosatum cum fumaria	*Rosa* spp., *Fumaria officinalis* L.	[8]
106	Oleum Amigda. dul.	*Prunus dulcis* (Miller) D.A. Webb	[53]
107	Syrupus Cichor. Com. Reub. Gul. -n.	*Cichorium intybus* L., *Rheum officinale* L.	[47,48]
108	Syrupus de s. Citri.	*Citrus limon* (L.) Osbeck	[44]
110	Syrupus Flor. Pers.	*Prunus persica* (L.) Batsch	[14,54,55]
111	Syrupus Diamor.	*Rubus ulmifolius* Schott	[13]
112	Oleum de. caparb. s.	*Capparis spinosa* L., *Olea europaea* L.	[9]
113	Pulvis Flor. Malu.	*Malus domestica* (Suckow) Borkh.	[14]
115	Syrupus ex. Trib. infus.	*Rosa* spp.	[10,11]
116	Syrupus Betonic.	*Stachys officinalis* (L.) Trevisan	[9]
117	Oxymel Simp.	*Vitis vinifera* L.	[56,57]
119	Mel. Ros. simp.	*Rosa* spp.	[58]
120	Aqua Flor. Cap.	*Capparis spinosa* L.	[59]
121	Aqua hed.terrest.	*Glechoma hederacea* L.	[60]
122	Aqua Matricar.	*Matricaria chamomilla* L., *Tanacetum parthenium* (L.) Sch. Bip.	[61,62,63]
			[61,64,65]
123	Aqua Melisse.	*Melissa officinalis* L.	[66,67]
124	Aqua Ruth. orten.	*Ruta graveolens* L.	[56,68]
126	Aqua Saponar.	*Saponaria officinalis* L.	[69,70]
127	Aqua Scorzon. nost.	*Scorzonera* spp.	[9]
128	Aqua Puv. arte.	*Artemisia* spp.	[9]
129	Aqua Scabiose.	*Knautia arvensis* (L.) Coult.	[9]
131	Aqua Artemis.	*Artemisia* spp.	[9]
132	Aqua Betton.	*Stachys officinalis* (L.) Trevisan	[71]
133	Aqua Petasit.	*Petasites hybridus* (L.) G. Gaertn., B. Mey. & Scherb.	[72]
134	Aqua Flor. samb.	*Sambucus nigra* L.	[73,74,75]
135	Aqua Tot. citri.	*Citrus medica* L.	[76,77]
136	Aqua Pimpinell.	*Pimpinella saxifraga* L.	[62]
137	Aqua Mil. fol.	*Achillea millefolium* L.	[62]
139	Aqua Card. bend.	*Centaurea benedicta* (L.) L.	[78]
140	Aqua Cent. min.	*Centaurium erythraea* Rafn.	[9]
141	Aqua Scabiose.	*Knautia arvensis* (L.) Coult.	[9]
142	Aqua Gland. persic.	*Prunus persica* (L.) Batsch, *Vitis vinifera* L.	[10,11]
143	Aqua Pulmon.	*Agrimonia eupatoria* L., *Borago officinalis* L., *Pulmonaria officinalis* L., *Rosa* spp., *Salvia officinalis* L., *Sanguisorba officinalis* L., *Symphytum officinale* L., *Veronica* spp.	[9]
144	Aqua Buglos.	*Anchusa officinalis* L.	[27,54]
145	Aqua Flor. lil. alb.	*Lilium candidum* L.	[54,61]
146	Aqua Fumar.	*Fumaria officinalis* L.	[8,47]
147	Flores Lil. Com.	*Lilium* spp.	[61]
149	Syrupus de Suc. cit.	*Citrus limon* (L.) Osbeck	[44]

**Table 2:** Categories of the pathologies treated with the 108 plant-based remedies, according to historical sources published between the XV and the XIX century [8,9,10,11,12,13,14,15,16,17,18,19,20,21,22,23,24,25,26,27,28,29,30,31,32,33,34,35,36,37,38,39,40,41,42,43,44,45,46,47,48,49,50,51,52,53,54,55,56,57,58,59,60,61,62,63,64,65,66,67,68,69,70,71,72,73,74,75,76,77,78]. The total number of vase-remedies used for the treatment of each category, along with the total number of plant-based ingredients in the remedies, are reported.

Category of Pathology	Tot. Vases Per Category	Tot. Species Per Category
Digestive tract disorders	46	83
General condition	30	59
Respiratory tract infections	28	69
Nervous system disorders	23	51
Skin diseases and traumas	21	45
Circulatory/lymphatic system disorders	21	76
Gynaecological disorders, obstetric and puerperal problems	18	38
Urinary tract disorders	14	33
Musculoskeletal system disorders and traumas	12	17
Other	10	32
Oropharyngeal cavity affections	2	9
Afflictions of the ear	2	3
Ophthalmic ailments	2	2

**Table 3:** List of species selected for the restoration project of the ancient *Hortus simplicium*.

***Adoxaceae***
1. *Sambucus nigra* L.
***Apiaceae***
2. *Carum carvi* L.
3. *Cuminum cyminum* L.
4. *Pimpinella anisum* L.
5. *Foeniculum vulgare* Mill.
***Boraginaceae***
6. *Borago officinalis* L.
***Compositae***
7. *Achillea millefolium* L.
8. *Centaurea benedicta* (L.) L.
9. *Cichorium intybus* L.
10. *Matricaria chamomilla* L.
***Cucurbitaceae***
11. *Citrullus colocynthis* (L.) Schrad.
12. *Ecballium elaterium* (L.) A. Rich.
***Cupressaceae***
13. *Juniperus communis* L.
***Cyperaceae***
14. *Cyperus esculentus* L.
***Euphorbiaceae***
15. *Euphorbia* spp.
***Gentianaceae***
16. *Centaurium erythraea* Rafn.
***Hypericaceae***
17. *Hypericum perforatum* L.
***Lamiaceae***
18. *Glechoma hederacea* L.
19. *Lavandula dentata* L.
20. *Melissa officinalis* L.
21. *Mentha aquatica* L.
22. *Mentha pulegium* L.
23. *Origanum majorana* L.
24. *Origanum dictamnus* L.
25. *Origanum vulgare* L.
26. *Rosmarinus officinalis* L.
27. *Salvia officinalis* L.
28. *Stachys officinalis* (L.) Trevis.
29. *Thymus* spp.
***Lauraceae***
30. *Laurus nobilis* L.
***Malvaceae***
31. *Althaea officinalis* L.
***Papaveraceae***
32. *Fumaria officinalis* L.
33. *Papaver somniferum* L.
***Plantaginaceae***
34. *Veronica* spp.
***Polygonaceae***
35. *Rumex conglomeratus* Murray
***Portulacaceae***
36. *Portulaca oleracea* L.
***Rosaceae***
37. *Malus domestica* Borkh.
38. *Rosa* spp.
***Saxifragaceae***
39. *Saxifraga* spp.
***Violaceae***
40. *Viola* spp.

## Data Availability

Not applicable.

## Supplementary Materials

The following are available online. Table S1: Results of the pharmacological survey in scientific literature.
