# Supplementary material for: Ca’ Granda, Hortus simplicium: Restoring an Ancient Medicinal Garden of XV–XIX Century in Milan (Italy)
Source: Molecules. 2021 Nov 17;26(22):6933. doi: 10.3390/molecules26226933 (PMC8620247; doi:10.3390/molecules26226933)
Supplement: Supplementary file 1 [file molecules-26-06933-s001.zip › molecules-1453946 - supplemetary updated.pdf]

Ca′ Granda, Hortus Simplicium: Restoring an Ancient Medicinal Garden of XV– XIX Century in Milan (Italy)

Pharmacological survey.

Table S1. Results of the pharmacological survey in scientific literature.

Species	Jar Inscription (Vase Number)	Plant Parts Historically Used	Historic Source	Therapeutic Use Obtained from the Historic Source	Bibliographic Reference	Mechanism of Action	Bibliographic Reference
**Acoraceae**							
Acorus calamus L.<br><br>*Calamo aromatico, sweet flag*	Electuarium Diacurcumae (**14**)<br><br>Electuarium bened. lax. (**19**)<br><br>Trochiscus d. Cappar. (**45**)<br><br>Electuarium d. Bac. laur. (**54, 80**)	Hypogeal parts	14: [12]<br><br>19: [16]<br><br>45, 54,80: [9]	Anti-inflammatory<br><br>Depurative/ Lymphatic system/Kidney stones<br><br>Diuretic<br><br>Gastrointestinal disorders (laxative, carminative)<br><br>Emmenagogue<br><br>Rubefacient, improves circulation<br><br>Respiratory tract<br><br>Thermogenic? Rubefacient?	Anti-asthma, hypotensive, antiarrhythmic, hypocholesterolemic: [86]<br><br>Antidiarrhoeal: [85]<br><br>Anti-inflammatory: [83,86]<br><br>Kidney stones, diuretic: [84]	Anti-inflammatory: decrease in the superoxide anion, total calcium levels, and myeloperoxidase (MPO) activity<br><br>Kidney stones: diuretic and nephroprotective actions	Anti-inflammatory: [83]<br><br>Kidney stones: [84]
**Adoxaceae**							
Sambucus ebulus L.<br><br>*Ebulo, dwarf elder*	Unguentum Agrippae (**7**)<br><br>Unguentum artanita (**24**)	Hypogeal parts Flowers	7: [10,11]<br><br>24: [20]	Antibacterial (Tuberculosis)<br><br>Anti-inflammatory<br><br>Circulatory system (circulation)	Antibacterial: [254,255,257]<br><br>Anti-inflammatory: [252,256,257]<br><br>Antioxidant: [252,257]<br><br>Diuretic:	Antibacterial: inhibits the production of δ-toxin by *Staphylococcus aureus*.<br><br>potent urease inhibitory activity	Antibacterial: [252,254]<br><br>Anti-inflammatory: [252]

				Depurative/ Lymphatic system<br><br>Diuretic	[253]<br><br>Soothing: [251]	of its flavonoids (specifically against *H. pylori*)<br><br>Anti-inflammatory: suppression of TNF-α, IL1-α, and IL1-β production. Interaction of ursolic acid interaction with COX-2 pathway	
Sambucus nigra **L.**<br><br>*Sambuco, European elder*	Electuarium Diascord. (**48**)<br><br>Reb. Sambuc. (**62**)<br><br>Roab. Sambuc. (**79**)<br><br>Aqua Flor. Samb. (**134**)	Leaves Flowers	48: [32]<br><br>48: [33]<br><br>62: [38,286]<br><br>79: [286]<br><br>134: [75]	Antipyretic<br><br>Antibacterial<br><br>Anti-inflammatory<br><br>Diaphoretic<br><br>Liver/Spleen disorders<br><br>Skin diseases (erysipelas)<br><br>Respiratory tract (mucus, expectorant, cold ecc)<br><br>Soothing	Diaphoretic: [259]<br><br>Respiratory system viral infections (Fruit): [258,260,261]<br><br>Soothing: [251]	Antiviral effects: Reduces hemagglutination and inhibit the replication of human influenza viruses. Decreases virus titer and inhibits viral protein synthesis or virus particle release.	Antiviral effects (Fruit): [260,261]
				**Agaricaceae**			
Agaricus bisporus (J.E. Lange) Imbach<br><br>or<br><br>***Agaricus campestris*** L.	Trochiscus de Agarici (**17**)<br><br>Pilulae Aloe. lota. (**23**)	Epigeal parts	[10,11,14]	Expectorant<br><br>Gastritis, stomach disorders	Antibacterial, antiviral, gastritis, stomach disorders, immunomodulant: [91,92]<br><br>Anti-inflammatory: [91]	Gastritis, stomach disorders: promotes digestion.	Gastritis, stomach disorders: [92]

Agarico, button mushroom, meadow mushroom							
**Anacardiaceae**							
Pistacia lentiscus L.<br><br>*Lentisco, mastic tree*	Unguentum Agrippae (**7**)<br><br>Oleum Mastyc (**31**)<br><br>Emplastru m crustae panis m. (**78**)<br><br>Pilulae Masticin. (**86**)	Exudate, Gum	7: [10,11]<br><br>31: [24]<br><br>78: [9]<br><br>86: [44]	Circulatory system (circulation) Gangrene: microcirculation/limbs circulatory disorders<br><br>Astringent<br><br>Expectorant, Lung pathologies, Tuberculosis<br><br>Digestive<br><br>Diuretic<br><br>Laxative	Anti-inflammatory, Antioxidant, Digestive: [222]<br><br>Kidney stones (Fruit): [223]	Kidney stones: decreases cell death induced by COM (Calcium oxalate monohydrate), decreases E-cadherin and H_2O_2 levels	Kidney stones (Fruit): [223]
Pistacia terebinthus L.<br><br>*Terebintina, Cyprus turpentine*	Pilulae de Amon. q. (**81**)	Resin	81: [42]	Antibacterial, Disinfectant (wounds)<br><br>Anti-inflammatory<br><br>Depurative<br><br>Diuretic<br><br>Expectorant, Respiratory tract<br><br>Laxative<br><br>Mucolytic<br><br>Scabies, Parasiticide	Antimicrobial (*Bacillus subtilis, Salmonella typhi, Escherichia coli, Staphylococcus epidermidis, Pseudomonas aeruginosa*, and *S. dysenteriae*), antioxidant, antiviral: [222]		

**Apiaceae**							
***Anethum graveolens* L.**<br><br>*Aneto, dill*	Unguentum Pectorale (**15**)	Fruit	15: [13]	Airways	Analgesic, antimicrobial, anti-inflammatory: [102]		
***Angelica* spp.**<br><br>*Angelica*	Electuarium Diascord. (**48**)	Hypogeal parts	48: [32, 33]	Antibacterial<br><br>Anti-inflammatory<br><br>Antipyretic<br><br>Diaphoretic<br><br>Expectorant/respiratory tract infections/pulmonary infections/antiviral	Antibacterial: [103] - *Angelica* spp (*i.e. A. archangelica* L. and *A. sinensis* (Oliv.) Diels))<br><br>Anti-inflammatory: [103] - *Angelica* spp (*i.e. A. sinensis* (Oliv.) Diels)<br><br>Antioxidant: [103]- *Angelica* spp (*i.e. A. archangelica* L. and *A. sinensis* (Oliv.) Diels)<br><br>Bronchodilator: [103] - *A. glauca* Edgew (Epigeal parts)		
Apium graveolens L.<br><br>*Sedano, celery*	Electuarium bened. lax. (**19**)	Seeds	19: [16]	Laxative			
Athamanta turbith (L.) Brot.<br><br>*Atamanta di Mattioli, candy carrot*	Electuarium bened. lax. (**19**)<br>Unguentum Artanita (**24**)	Hypogeal parts	19: [16]<br><br>24: [20]	Emetic<br><br>Emmenagogue<br><br>Laxative<br><br>Vermifuge/Anthelmintic/Antibacterial	Antimicrobial: [109]		
Carum carvi L.	Electuarium	Fruit	19: [16]<br>54,80: [9]	Diuretic<br><br>Emmenagogue	Dyspepsia, diuretic: [119]	Emmenagogue:	Emmenagogue: [119]

Cumino dei prati, caraway	bened. lax. (**19**)<br><br>Electu-arium d. Bac. laur. (**54,80**)			Gastrointestinal disorders (laxative, carminative)	Emmenagogue, gastrointestinal disorders: [118,119]	increases the estrogen reduces the progestrone and FSH levels at proestrus phase<br><br>Presence of luteolin and apigenin (estrogenic isoflavonoids)<br><br>Gastrointestinal disorders: spasmolytic activity.	Gastrointestinal disorders: [118,119]
Cuminum cynimum L.<br><br>*Cumino, cumin*	Trochiscus de Mirra (**51**)<br><br>Electu-arium d. Bac. laur. (**54,80**)	Leaves Fruit	51,54,80: [9]	Diuretic<br><br>Emmenagogue, facilitates birth (Increases the contractions), Reduce menopausal flushing<br><br>Gastrointestinal disorders (carminative)	Emmenagogue, gastrointestinal disorders: [118]	Gastrointestinal disorders: increases acid secretion by a cholinergic mechanism. Increases amylase, protease, lipase, and phytase activities	Gastrointestinal disorders: [118]
Daucus carota L.<br><br>*Carota, carrot*	Electu-arium Diacurcumae (**9**)	Hypogeal parts	[12]	Airways problems (non-specified)<br><br>Gastrointestinal disorders (non-specified)<br><br>Rubefacient, Circulatory problems (non-specified)<br><br>Thermogenic	Circulation: [154]	Circulation: Antioxidant properties of phenolic compound minimise the risk of cardiovascular diseases Hypocholesterolemic, hypolipidemic, and hypotensive properties. Antiplatelet aggregatory characteristics of polyacetylenes	Circulation: [154]

Dorema ammoniacum D. Don<br><br>*Gomma ammoniaca, ammoniacum gum*	Trochiscus d. Cappar. (**45**)<br><br>Pilulae de Amon. Q. (**81**)	Gum	45: [9]<br><br>81: [42]	Antibacterial, Disinfectant (wounds)<br><br>Anti-inflammatory<br><br>Antiviral<br><br>Kidney stones<br><br>Decongestant<br><br>Depurative<br><br>Dermatitis, Allergies<br><br>Diuretic<br><br>Emmenagogue<br><br>Expectorant, Respiratory tract<br><br>Laxative<br><br>Scabies (Parasiticide)	Analgesic, Anti-inflammatory, neuroprotector: [157]<br><br>Antibacterial: [155,158]<br><br>Antiseptic (ulcers), kidney stones, depurative, dermatitis, diuretic, laxative [156]<br><br>Antiviral, respiratory system: [158]
Eryngium maritimum L.<br>*Eringio marittimo, sea holly*	Unguentum Agrippae (**7**)	Hypogeal parts	[10,11]	Circulatory system (circulation)<br><br>Edema, extravascular fluid accumulation<br><br>Diuretic	Antioxidant (circulation): [161]
Ferula gummosa Boiss.<br><br>*Galbano, galbanum*	Pilulae de Amon. q. (**81**)	Gum	81: [42]	Antibacterial, Disinfectant (wounds)<br><br>Anti-inflammatory<br><br>Decongestant<br><br>Depurative<br><br>Dermatitis, Allergies<br><br>Diuretic	Antibacterial: [166]<br>[168] - Seeds E.O.<br><br>Anti-inflammatory: [294]

				Expectorant, Respiratory tract<br><br>Laxative<br><br>Scabies (Parasiticide)			
Ferula persica Willd.<br><br>*Segapeno, segapenum*	Unguentum artanita (**24**)	Epigeal parts	[20]	Emetic<br><br>Laxative<br><br>Vermifuge			
Foeniculum vulgare Mill.<br><br>*Finocchio, fennel*	Electuarium bened. lax. (**19**)<br><br>Syrupus d. Duab. rad. (**33**)<br><br>Syrupus d. Artemisie q.p. (**96**)	Hypogeal parts<br>Fruit	19: [16]<br><br>33: [25]<br><br>96: [13,19]	Blood depurative<br><br>Effects on Nervous System<br><br>Expectorant<br><br>Laxative	Antimicrobial, blood depurative:<br>[169]	Blood depurative:<br><br>β-Myrcene, Limonene: Decreases AST, ALT, ALP, and bilirubina serum levels<br><br>*Trans*-Anethole: antithrombotic.<br><br>5-Methoxypsoralen: inhibits cytochrome P450-3A4.<br><br>Essential oil: hypoglycemic	Blood depurative:<br>[169]
Narthex asafoetida Falc. ex Lindl.<br><br>*Sin:* ***Ferula asafoetida*** H. Karst.	Pilulae Fetid. (**46**)	Resin	[31]	Spasmolytic	Spasmolytic:<br>[201,202]	Spasmolytic: azulene, ferulic acid, luteolin, umbelliferone, valeric acid.<br><br>Activity on muscarinic receptors (tested on tracheal smooth muscle)	Spasmolytic:<br>[201,202]

Finocchio Fetido, asafetida							
Petroselinum crispum (Mill.) Fuss<br><br>*Prezzemolo, parsley*	Syrupus d. Duab. rad. (**33**)<br><br>Syrupus d. Artemisie q.p. (**96**)	Hypogeal parts<br>Fruit (Seeds)	33: [25]<br>96: [13]<br><br>96: [19]	Blood depurative<br><br>Expectorant<br><br>Effects on Nervous System	Antioxidant, diuretic: [215]	Antioxidant: dose dependent inhibition of lipid peroxidation, dose dependently hydroxyl radical scavenging, DPPH radical scavenging;<br><br>Diuretic: Significant increase in urine volume; significant decreas in activity of kidney cortex and medulla Na^+-K^+ ATPase; significant increase in kidney urine flow rate	[215]
Pimpinella anisum L.<br><br>Anice, anise	Electuarium Diacurcumae (**9**)<br><br>Conserva Hamech (**14**)	Fruit	9: [12]<br><br>14: [9]	Skin diseases: Scabies and Scall (Parasiticide), Itch, and irritations<br><br>Airways (non-specified)<br><br>Gastrointestinal disorders (non-specified)<br><br>Rubefacient, Circulation<br><br>Scurvy<br><br>Syphilis (cutaneous symptoms only)	Antimicrobial, antioxidant, airways, gastrointestinal disorders: [216]	Airways: muscle relaxant of tracheal chain<br><br>Antiulcer: significantly inhibits gastric mucosal damage induced by necrotizing agents and indomethacin.<br><br>Laxative: (with *Foeniculum vulgare*, *Sambucus nigra*, and *Cassia*	[216]

				Thermogenic		*augustifolia*) decreases colonic transit time; increases the number of daily evacuations	
***Pimpinella saxifraga* L.** *Pimpinella sassifraga, burnet-saxifrag*	Aqua Pimpinell. (**136**)	Hypogeal parts	136: [62,285,286]	Antibacterial (specifically oropharyngeal cavity)<br><br>Anti-inflammatory<br><br>Antiviral<br><br>Expectorant	Antibacterial: [217]		
				**Aristolochiaceae**			
Aristolochia rutunda L. *Aristolochia rotonda, round-leaved birthwort*	Trochiscus d. Cappar. (**45**)	Hypogeal parts	45: [9]	Anti-inflammatory<br><br>Kidney stones<br><br>Diuretic<br><br>Emmenagogue			
Asarum europaeum L. *Asaro, European wild ginger*	Trochiscus Absynthi (**11**)<br><br>Syrupus d. Artemisie q.p. (**96**)	Hypogeal parts Seeds	11: [9]<br><br>96: [13,19]	Aperitive, digestive<br><br>Anti-inflammatory<br><br>Choleretic<br><br>Blood depurative<br><br>Effects on Nervous System	Anti-Alzheimer: [106] Antitumoral: [80] - Aerial parts	Anti-Alzheimer: neuroprotectivity against β-amiloyd plaques caused neurotoxicity by inhibiting the effects of NO overproduction in the hippocampus.<br><br>*α*-asarone: positive effects on neuroinflammation, onset of clonic and/or tonic seizures; brain antioxidant enzymes	Anti Alzheimer: [106]

						β-asarone: neuroprotective action; positive effects on memory and learning; improves brain microcirculation	
**Asparagaceae**							
Asparagus officinalis L.<br><br>Asparago, asparagus	Electuarium bened. lax. (**19**)	Pulp from the female plant	19: [16]	Laxative	Laxative: [107]	Laxative: aerial parts facilitate evacuation increasing fecal bulk with undigested fiber	Laxative: [107]
Drimia maritima (L.) Stearn<br><br>*Cipolla di mare, sea squill*	Unguentum Agrippae (**7**)<br><br>Oxymel Scyll. (**27**)	Hypogeal parts Epigeal parts	7: [10,11]<br><br>27: [22]	Circulatory system (circulation)<br><br>Edema, extravascular fluid accumulation<br><br>Diuretic<br><br>Emetic<br><br>Expectorant	Antimicrobial, Antioxidant, antitumoral, circulatory system: [159]	Circulatory system:<br><br>Scillonin: digital-like effects	Circulatory system: [159]
Ruscus aculeatus L.<br><br>Rusco, butcher's broom	Electuarium bened. lax. (**19**)	Seeds, Pulp	19: [16]	Laxative	Spasmolytic: [235] - Aerial parts		
**Aspleniaceae**							
Asplenium scolopendrium L.<br><br>*Scolpendra comune, hart's*	Forse Electuarium Diacurcumae (**9**)<br><br>Trochiscus d. Cappar. (**45**)	Leaves	9: [12]<br><br>45: [9]	Anti-inflammatory<br><br>Kidney stones<br><br>Diuretic<br><br>Emmenagogue<br><br>Airways (non-specified)	Antioxidant: [108]		

tongue fern				Gastrointestinal disorders (non-specified)<br><br>Rubefacient, Circulation<br><br>Thermogenic			
Ceterach officinarum Willd.<br><br>*Cedracca, rustyback*	Forse Electuarium Diacurcumae (**9**)	Leaves	[12]	Airways (non-specified)<br><br>Gastrointestinal disorders (non-specified)<br><br>Rubefacient, Circulation<br><br>Thermogenic	Antioxidant: [127,128]		
				**Boraginaceae**			
Anchusa officinalis L.<br><br>*Buglossa, common bugloss*	Aqua Buglos. (**144**)	Aerial parts	[27]	Analgesic<br><br>Anti-depressant<br><br>Antipyretic<br><br>Kidney stones<br><br>Diuretic<br><br>Circulatory system<br><br>Emmenagogue<br><br>Facilitate birth (Increase the contractions)<br><br>Skin diseases: erysipelas<br><br>Tonic	Anti-diabetic (specifically circulation), anti-inflammatory, antioxidant: [101]	Anti-inflammatory: inhibits LOX and HYA	[101]
Borago officinalis L.<br><br>*Borragine, common borage*	Conserva Boragina. (**21**)	<u>Epigeal parts</u><br><u>Flowers</u>	21: [17]<br><br>21: [18]<br><br>143: [9]	Anti-depressant<br><br>Circulatory system<br><br>Respiratory system	Anti-asthma: [112,113] - <u>Leaves</u><br><br>Anti-inflammatory: [111] - <u>Seeds</u>	Anti-asthma: Ca^{2+} antagonist activity.<br><br>Spasmolytic: Ca^{2+} antagonist activity.	Anti-asthma, spasmolytic, circulatory system: [112]

	Aqua Pulmon. (**143**)			Tonic	Antioxidant: [110]<br>Circulatory system: [110, 112] - <u>Leaves</u><br>Spasmolytic: [112]- <u>Leaves</u>	Circulatory system: Ca^{2+} antagonist activity.<br>Anti-inflammatory: suppresses TNF-α and PGE2 production.	Anti-inflammatory: [111]
Cynoglossum officinale L.<br>*Cinoglossa, hound's tongue*	Pilulae de Cinoglo. (**69**)	Hypogeal parts	[9,285,286]	Astringent for gums<br>Expectorant<br>Sedative-hypnotic	Analgesic, antibacterial, antihemorrhagic, anti-inflammatory, antiseptic: [152]		
Pulmonaria officinalis L.<br>*Polmonaria, Jerusalem-sage*	Aqua Pulmon. (**143**)	Buds	[9]	Circulatory system	Antioxidant: [234]	Antioxidant: polyphenols, flavones, and proanthocyanidins have antioxidant and antiradical activity.	Antioxidant: [234]
Symphytum officinale L.<br>*Consolida maggiore, common comfrey*	Aqua Pulmon. (**143**)	Hypogeal parts	[9]	Circulatory system	Anti-inflammatory: [269]	Anti-inflammatory: impairs the interleukin-1 (IL-1) induced expression of pro-inflammatory markers including E-selectin, VCAM1, ICAM1, and COX-2. Inhibits the activation of NF-κB.	Anti-inflammatory: [269]
				**Burseraceae**			
Boswellia serrata	Unguentum Citrini (**44**)	Gum-resin ottenuta	44: [30]<br>69: [9]	Astringent for gums<br>Expectorant	Antibacterial, antifungal, Anti-inflammatory,		

Roxb. ex Colebr.<br><br>*Boswellia, Indian olibanum tree*	Pilulae de Cinoglo. (**69**)	dall'incisione del tronco		Skin blemishes (scars, freckels, irritations)<br><br>Sedative-hypnotic	Antioxidant, Sedative: [114]<br><br>Antimicrobial, wounds: [116]<br><br>Asthma: [295]		
Commiphora gileadensis (L.) C.Chr.<br><br>*Balsamo arabo, Balsam of Gilead*	Electuarium Diacurcumae (**9**)	Fruit, balsamo	9: [12]	Airways (non-specified)<br><br>Gastrointestinal disorders (non specificati)<br><br>Thermogenic	Antibacterial: [139]		
Commiphora myrrha (Nees) Engl.<br><br>*Mirra, Myrrh*	Electuarium Diacurcumae (**9**)<br><br>Unguentum artanita (**24**)<br><br>Trochiscus de Mirra (**51**)<br><br>Pilulae de Cinoglo. (**69**)<br><br>Pilulae de Amon. q. (**81**)	Gum, Gum-resin	9: [12]<br><br>24: [20]<br><br>51: [285,286]<br><br>51, 69: [9]<br><br>81: [42]	Antibacterial, Disinfectant (wounds)<br><br>Anti-inflammatory<br><br>Astringent for gums<br><br>Depurative<br><br>Emmenagogue<br><br>Expectorant<br><br>Facilitate birth (Increase the contractions)<br><br>Sedative-hypnotic<br><br>Airways (non-specified), antihistamine<br><br>Gastrointestinal disorders (Stomach depurative, laxative)	Antibacterial, antifungal, anti-inflammatory, antiseptic (wounds), digestive (dyspepsia and gastritist), emmenagogue, stimulant of the uterus (abortive), gingivitis, respiratory system disorders: [140]<br><br>Antioxidant: [142]<br><br>Antihistamine,gingivitis: [141]	Antioxidant: scavenging activity on free radicals.<br><br>Anti-inflammatory and antihistamine: inhibit the expression and release of IL-31 in HMC-1 cells. Inhibits the production of prostaglandin E2, nitrous oxide, and pro-inflammatory cytokines including tumor necrosis factor (TNF)-α, IL-6, and IL-8 on human gingival fibroblasts. Flavonoids, sesquiterpenes, terpenoids, and eugenol	Antioxidant: [142]<br><br>Anti-inflammatory and antihistamine: [141]

				Menopausal flushing<br><br>Scabies (Parasiticide)<br><br>Thermogenic<br><br>Vermifuge		suppress MAPK and NF-κB activation.<br>Inhibits histamine release in activated HMC-1 cells.	
				**Capparaceae**			
Capparis spinosa L.<br><br>*Cappero, caper bush*	Unguentum artanita (**24**)<br><br>Trochiscus d. Cappar. (**45**)<br><br>Oleum de. caparb. s. (**112**)<br><br>Aqua Flor. Ca (**120**)	Hypogeal parts, scorza della Hypogeal parts	24: [20]<br><br>45, 112: [9]<br><br>112: [285,286]<br><br>120: [59]	Antibacterial (Tuberculosis)<br><br>Anti-inflammatory<br><br>Depurative<br><br>Diuretic<br><br>Emmenagogue<br><br>Liver (hepatoprotector etc.)<br><br>Stomach-ache (Spasmolytic)	Antimicrobial, anti-inflammatory, hypocholesterolemic (Fruit): [122]<br><br>Hepatoprotector: [121,122] - Aerial parts<br><br>Hypoglycemic, hypolipidemic: [120] - Fruit		
				**Caprifoliaceae**			
Knautia arvensis (L.) Coult.<br><br>*Scabiosa, field scabious*	Aqua Scabiose. (**129, 141**)	Epigeal parts	129, 141: [9,285,286]	Antipyretic			
Valeriana spp<br><br>*Valeriana celtica, Valeriana selvatica, Alpine valerian, Valerian*	Electuarium Diacurcumae (**9**)	Hypogeal parts	[12]	Airways (non-specified)<br><br>Gastrointestinal disorders (non-specified)<br><br>Thermogenic	Gastrointestinal disorders: [273]	Antidiarrhoeal effects: block of calcium channels	Antidiarrhoeal effects: [273]
				**Caryophyllaceae**			

Dianthus caryophyllus L. *Garofano commune, carnation*	Electuarium bened. lax. (**19**)	Flowers	[16]	Laxative			
Saponaria officinalis L. *Saponaria, common soapwort*	Aqua Saponar. (**126**)	Hypogeal parts Leaves	[70]	Aperitive, digestive<br><br>Anti-inflammatory<br><br>Antiviral<br><br>Depurative<br><br>Myorelaxant<br><br>Venereal diseases and Syphilis (cutaneous and musculoskeletal symptoms only)	Antiviral: [265]	Antiviral (against plants viruses): possible antiviral activity mediated by some RIPs (Ribosome-inactivating proteins) such as PAP. RIPs may be suicidal agents released from cell walls or vacuoles against viruses.	Antiviral: [265]
				**Compositae**			
Achillea millefolium L. *Millefoglio, common Yarrow*	Electuarium bened. lax. (**19**)<br><br>Electuarium Diascord. (**48**)<br><br>Aqua Mil. fol. (**137**)	Aerial parts Seeds	19: [16]<br><br>19, 48: [32]<br><br>48: [33]<br><br>137: [62]	Antibacterial<br><br>Anti-inflammatory<br><br>Antipyretic<br><br>Kidney stones<br><br>Diaphoretic<br><br>Digestive<br><br>Disinfectant (wounds)<br><br>Diuretic<br><br>Effects on autonomic nervous system (vagus)	Antibacterial, Antiulcer, Emmenagogue: [82]	Emmenagogue: estrogenic activity of apigenin and luteolin.<br><br>Antiulcer: protects the gastric mucosa against acute gastric lesions and promotes regeneration of the gastric mucosa. Inhibits gastric secretion or increases protective factors.	Emmenagogue, antiulcer: [82]

				Emmenagogue<br><br>Hemorrhoids<br><br>Expectorant<br><br>Laxative			
***Artemisia absinthium* L.**<br><br>*Assenzio, wormwood*	Electuarium Diacurcumae (**9**)<br><br>Trochiscus Absÿnthi (**11**)<br><br>Conserva Hamech (**14**)<br><br>Oleum Absinthÿ (**34**)<br><br>Conserva Absinth. (**64**)<br><br>Syrupus Absynthii (**92**)	Leaves<br>Flowering aerial parts<br>Fruit<br>Seeds	9: [12]<br><br>11, 14: [9]<br><br>34: [26]<br><br>64: [39,40]<br><br>92: [45,46]	Airways (non-specified)<br><br>Anti acidic<br><br>Anticonvulsant<br><br>Antiemetic<br><br>Anti-inflammatory<br><br>Antipyretic<br><br>Aperitive, digestive<br><br>Choleretic<br><br>Diuretic<br><br>Gastrointestinal disorders (acidic reflux)<br><br>Jaundice<br><br>Kidney stones<br><br>Parasiticide (parasitosis that causes intermittent fever, i.e., malaria)<br><br>Rubefacient, Circulation<br><br>Scurvy<br><br>Skin diseases: Scabies (Parasiticide), Scall, Itch, and irritations	Antibacterial, anthelmintic, antifungal, antiprotozoal, antiviral, antioxidant, anti-inflammatory, antipyretic, analgesic, antiulcer, immunomodulatory effects, jaundice: [104]	Anthelmintic: α- and β-thujone: decrease juvenile larval motility and egg development of *Ascaris suum*<br><br>Antibacterial: Essential oil: suppreses biosynthesis of proteins, RNA, DNA, and polysaccharides in bacterial walls.<br><br>Anti-inflammatory: 5,6,3′,5′-tetramethoxy 7,4′-hydroxyflavone, cardomonin, caruifolin D: suppression of pro-inflammatory mediators (iNOS, PGE, NO, COX-2, NF-kB).<br><br>Antioxidant: Phenolic compounds and flavonoids: free radical scavengers	[104]

	Syphilis (cutaneous symptoms only)<br><br>Thermogenic<br><br>Vermifuge	Reduction of lipid peroxidation, decreasing of TBARS level, and recovery of endogenous antioxidant (SOD, GSH).<br><br>Antipyretic and analgesic: 22-dien-3 Bat, 24-β-ethyl p-cholesta-7: nicotinic and muscarinic action.<br><br>Antiprotozoal: Essential oil, flavonoids, artemisin: stimulate both heme and mitochondrial-mediated degradation cascade; inhibit PfATP6, LDH, SAMS, PyrK, SpdSyn, OAT enzyme activities<br><br>Antiviral: inhibits the HIV-1 integrase enzyme from connecting the host cell DNA with the reversibly transcribed viral DNA<br><br>Digestive: Bitter substances, essential oils:	

						enhance bile production and secretion.<br><br>Immunomodulatory effects: Polysaccharides: initiate Th1 response and activate NO synthesis.<br><br>Jaundice, hepatitis: hepatoprotective activity, liver microsomal drug-metabolizing enzymes suppression, free radical scavenging activity, calcium channels blockage.	
***Artemisia** spp*<br><br>*Artemisia*	Syrupus d. Artemisie q.p. (**96**)<br><br>Aqua Puv. arte. (**128**)<br><br>Aqua Artemis. (**131**)	Aerial parts	96: [13,19]<br><br>128, 131: [9]	Aperitive, Digestive<br><br>Depurative (del sangue)<br><br>Effects on Nervous System<br><br>Menopausal flushing<br><br>Vermifuge	Hepatoprotector: [105] - Leaves	Hepatoprotector: decreases α-FP, γ-GT, 5′-NT, ALT, AST, LD, and G6PD levels; decreases total bilirubin levels; increases albumin levels.	Hepatoprotector: [105]
Centaurea benedicta (L.) L.<br><br>*Cardo santo, blessed thistle*	Electuarium Diascord. (**48**)<br><br>Aqua Card. bend. (**139**)	Flowering aerial parts	48: [32,33]<br><br>139: [78]	Antibacterial<br><br>Antipyretic<br><br>Antiviral<br><br>Diaphoretic<br><br>Disinfectant (wounds)	Anti-schistosomiasis (fever as one of the symptoms): [126]<br><br>Wounds and ulcers: [125]		

				Respiratory system diseases			
***Cichorium endivia* L.** *Endivia, endive*	Diatrium. santal. (**50**)	Epigeal parts	[9]	Anti-inflammatory Depurative Tonic	Antioxidant: [130]-Hypogeal parts Hepatoprotector: [129]		
***Cichorium intybus* L.** Cicoria, cichory	Syrupus Cichor. Com. Reub. Gul. -n. (**94, 107**)	Hypogeal parts	94, 107: [47,48,285, 286]				
***Eupatorium cannabinum* L.** *Canapa acquatica, hemp-agrimony*	Electuarium Diacurcumae (**9**)	Flowering aerial parts	[12]	Airways (non-specified) Gastrointestinal disorders (acidic reflux) Thermogenic	Anti-inflammatory: [162] Choleretic, Hepatoprotector: [163]	Anti-inflammatory: suppresses fMet-Leu-Phe/cytochalasin B (fMLP/CB)-induced superoxide anion generation and elastase release	Anti-inflammatory: [162]
***Matricaria chamomilla* L.** *Camomilla, chamomile*	Unguentum artanita (**24**) Aqua Matricar. (**122**)	Flowering aerial parts	24: [20] 122: [61,62]	Stomach depurative Laxative Spasmolytic Vermifuge	Antidiarrhoeal: [79] Antiulcer, gastrointestinal disorders, spasmolytic: [190]		
***Petasites hybridus* (L.) G.Gaertn., B.Mey. & Scherb.** *Petasite, butterbur*	Aqua Petasit. (**133**)	Hypogeal parts Leaves	[72]	Antipyretic Antiviral Aperitive, digestive Diaphoretic Diuretic Emmenagogue Expectorant	Antiulcer, expectorant: [214]	Antiulcer: inhibition of lipoxygenase activity and leukotriene biosynthesis Expectorant: Petasin: antispasmodic properties. Improves lung ventilation and	Antiulcer, expectorant [214]

						reduces bronchial reactivity.	
Rhaponticum scariosum Lam.<br><br>*Fiordaliso rapontico, giant scabiosa*	Diatrium. santal. (**50**)	Flowers	[9]	Anti-inflammatory<br><br>Depurative<br><br>Tonic			
***Scorzonera* spp.**<br><br>*Scorzonera*	Electuarium Diascord. (**48**)<br><br>Aqua Scorzon. nost. (**127**)	Epigeal parts	48: [32,33]<br><br>127: [9]	Antibacterial<br><br>Antipyretic<br><br>Disinfectant (wounds)<br><br>Diaphoretic<br><br>Respiratory system diseases	Antibacterial: [266,268,296]<br><br>Antimicrobial, antifungal, anti-inflammatory, antinematodi: [296]<br><br>Wounds: [267,296]		
Tanacetum parthenium (L.) Shc.bip.<br><br>*Tanaceto , feverfew*	Aqua Matricar. (**122**)	Leaves	122: [61,64,65]	Analgesic<br><br>Anti-inflammatory<br><br>Antipyretic<br><br>Aperitive, digestive<br><br>Emmenagogue<br><br>Facilitates birth (Increases the contractions)<br><br>Spasmolytic<br><br>Vermifuge	Analgesic: [270,271]<br><br>Anti-inflammatory, Spasmolytic: [270]	Anti-inflammatory: Parthenolide: inhibits production of inflammatory prostaglandins.<br><br>Spasmolytic: Sesquiterpene lactones: possibly due to inhibition of the influx of extracellular calcium into vascular smooth muscle cells	Anti-inflammatory, spasmolytic: [270]
				**Convolvulaceae**			
Convolvulus	Electuarium bened. lax. (**19**)	Hypogeal parts, juice Fruit	19: [16]<br><br>24: [20]	Antibacterial, Disinfectant (wounds)			

scammonia L.<br><br>*Gialappa, scammony*	Unguentum artanita (**24**)<br><br>Unguentum Diagridium (**68**)<br><br>Pilulae de Amon. q. (**81**)		81: [42]	Anti-inflammatory<br><br>Stomach depurative<br><br>Diuretic<br><br>Expectorant<br><br>Laxative<br><br>Scabies (Parasiticide)<br><br>Vermifuge	
**Cucurbitaceae**					
Bryonia *spp*<br><br>*Brionia, bryony*	Unguentum Agrippae (**7**)	Hypogeal parts	[10,11]	Anti-inflammatory<br><br>Diuretic<br><br>Edema, extravascular fluid accumulation<br><br>Circulatory system	Anti-inflammatory: [117]
Citrullus colocynthis (L.) Schrad.<br><br>*Coloquintide, bitter apple*	Trochiscus Alhandal (**4**)<br><br>Conserva Hamech. (**14**)<br><br>Unguentum artanita (**24**)<br><br>Electuarium Diaccatol. (**41**)<br><br>Trochiscus	Fruit<br>Fruit, epicarpo<br>Seeds	4, 14, 57: [9]<br><br>24: [20]<br><br>41: [29]	Anxiolytic, antidepressant<br><br>Anti-inflammatory<br><br>Antihistamine<br><br>Stomach depurative<br><br>Expectorant<br><br>Laxative<br><br>Skin diseases: Scabies (Parasiticide), Scall, Irritations<br><br>Scurvy	Anti-inflammatory: [138]

	Aland. (**57**)			Syphilis (cutaneous symptoms only)			
Cucumis melo L.<br>*Melone, melon*	Di-atrium. santal. (**50**)	Seeds	[9]	Anti-inflammatory<br>Depurative<br>Lymphatic system<br>Tonic	Anti-inflammatory: [147]	Anti-inflammatory: suppresses PGE-2, TNF-α, IL-6, and IL-1β production.	Anti-inflammatory: [147]
Ecballium elaterium (L.) A. Rich.<br>*Cocomero asinino, squirting cucumber*	Unguentum Agrippae (**7**)<br>Unguentum artanita (**24**)	Hypogeal parts<br>Fruit	7: [10,11]<br>24: [20]	Stomach depurative<br>Diuretic<br>Edema, extravascular fluid accumulation<br>Laxative			
				**Cupressaceae**			
Juniperus communis L.<br>*Ginepro, common juniper*	Syrupus d. Artemisie q.p. (**96**)	Galbuli ('bacche')	[13,19]	Blood depurative<br>Reinforces nervous system			
Juniperus sabina L.<br>*Sabina, savin*	Syrupus d. Artemisie q.p. (**96**)	Non specificato	[13,19]	Blood depurative<br>Reinforces nervous system			
				**Cyperaceae**			
Cyperus esculentus L.<br>*Zigolo, yellow nutgrass*	Electuarium Diacurcumae (**9**)<br>Trochiscus d. Cappar. (**45**)	Hypogeal parts	9: [12]<br>45: [9]	Anti-inflammatory<br>Depurative<br>Diuretic<br>Emmenagogue<br>Airways (non-specified)	Anti-inflammatory: [153]		

				Gastrointestinal disorders (non-specified)<br><br>Thermogenic			
**Dryopteridaceae**							
Dryopteris filix-mas (L.) Schott<br><br>*Felce maschio, male fern*	Unguentum artanita (**24**)	Hypogeal parts, Resin	[20]	Antibacterial (Tuberculosis)<br><br>Anti-inflammatory<br><br>Depurative	Anti-inflammatory: [160]	Anti-inflammatory: inhibits acute phase of inflammatory responses (histamine, serotonin, prostaglandins, and bradykinin).	Anti-inflammatory: [160]
**Equisetaceae**							
Equisetum arvense L.<br><br>or<br><br>***Equisetum hyemale*** L.<br><br>*Equiseto, horsetail*	Aqua aequi (**3, 16**)	Fusti Leaves	[8]	Gastric ulcer			
**Euphorbiaceae**							
Euphorbia esula L. *Esula, leafy spurge*	Electuarium bened. Lax(**19**)	Hypogeal parts Epigeal parts	[16]	Laxative			
Euphorbia spp<br><br>*Euforbio, spurge*	Unguentum artanita (**24**)	Epigeal parts	[20]	Laxative<br><br>Stomach depurative<br><br>Vermifuge	Laxative: [165]<br><br>Vermifuge/Anthelmintic: [164]	Laxative: increases mRNA and protein expressions of aquaporin 3 and 4 in mucosal epithelial cells in the colon	Laxative: [165]
**Gentianaceae**							

Centaurium erythraea Rafn.<br><br>*Centaurea minore, European centaury*	Syrupus d. Artemisie q.p. (**96**)<br><br>Aqua Cent. Min. (**140**)	Flowering aerial parts Leaves	96: [13,19]<br><br>140: [9]	Antipyretic<br><br>Antiviral<br><br>Blood depurative<br><br>Liver (hepatoprotector, etc.)<br><br>Reinforces nervous system	Antipyretic: [123]<br><br>Hepatoprotector: [124]		
			**Hypericaceae**				
Hypericum perforatum L.<br><br>*Iperico, St. John's wort*	Oleum Hyperic. q.pl. (**104**)	Flowering aerial parts	[52,285,286]	Antibacterial, disinfectant (wounds)<br><br>Local anaesthetic<br><br>Anti-inflammatory<br><br>Otitis	Antibacterial: [180]<br><br>Anti-inflammatory: [179]	Anti-inflammatory: inhibits 5-lipoxygenase. Inhibits human inducible nitric oxide synthase (iNOS) mRNA, protein, and nitric oxide (NO) production	Anti-inflammatory: [179]
			**Iridaceae**				
Crocus* spp.**<br><br>or<br><br>Crocus sativus*** L.<br><br>*Croco, zafferano, saffron*	Electuarium Diacurcumae (**9**)<br><br>Electuarium bened. lax. (**19**)<br><br>Pilulae de Cinoglo. (**69**)	Flowers, solo pistilli	9: [12]<br><br>19: [16]<br><br>69: [9]	Astringent for gums<br><br>Expectorant<br><br>Sedative-hypnotic<br><br>Laxative<br><br>Myorelaxant<br><br>Airways (non-specified)<br><br>Gastrointestinal disorders (non-specified)<br><br>Thermogenic	Gastrointestinal disorders: [143,145]<br><br>Airways: [144]<br><br>Sedative: [146]	Gastrointestinal disorders (ulcer): prevents gastric mucosa damage (enriches glutathione levels, reduces lipid peroxidation).<br><br>Airways: *C. sativus:* reduces NO, iNOS, and inflammatory cytokines levels in lung tissues. Saffron and kaempferol: reduce the production of NO, and	Gastrointestinal disorders: [143]<br><br>Airways: [144]

						inflammatory mediators in the serum and broncho-alveolar lavage fluid.<br>Increase anti-inflam-matory medi-ators.<br>Smooth mus-cle relaxant effects on tra-cheal smooth muscle (stim-ulate β-adre-noceptor and inhibit musca-rinic recep-tors).	
Iris tu-berosa L.<br><br>*Sin:* ***Hermo-dactylus tu-berosus*** (L.) Mill.<br><br>*Ermo-datili, snake's-head*	Electu-arium bened. lax. (**19**)<br><br>Pulvis her-modac. (**82**)	Hypogeal parts	19: [16]<br><br>82: [43]	Anti-inflamma-tory<br><br>Laxative			
			**Lamiaceae**				
Clino-podium nepeta subsp. ***glandu-losum*** (Req.) Govaert s<br>*Sin:* ***Cal-amintha offici-nalis*** Moench	Tro-chiscus d. Cap-par. (**45**)	Flower-ing aerial parts	[9]	Anti-inflamma-tory<br><br>Kidney stones<br><br>Diuretic<br><br>Emmenagogue			

Nepeta, lesser calamint							
Glechoma hederacea L.<br><br>*Edera terrestre, ground ivy*	Syrupus heder. terres. (**35**)<br><br>Extractus haed. terrest. (**69**)<br><br>Extractus haed. terrest. (**75, 87**)<br><br>Aqua hed.terrest. (**121**)	Epigeal parts	35: [27,285,286]<br><br>69: [41,285,286]<br><br>75, 87: [285,286]<br><br>121: [60,285,286]	Anti-inflammatory<br><br>Antitussive<br><br>Cephalea<br><br>Wound healing<br><br>Choleretic<br><br>Diaphoretic<br><br>Diuretic<br><br>Emmenagogue<br><br>Jaundice	Anti-inflammatory: [173]	Anti-inflammatory: Inhibits NO, iNOS, and COX-2.	Anti-inflammatory: [173]
Lavandula dentata L.<br><br>*Spigonardo, French lavender*	Electuarium Diacurcumae (**9**)<br><br>Trochiscus Absynthi (**11**)<br><br>Electuarium bened. lax. (**19**)<br><br>Unguentum artanita (**24**)<br><br>Oleum. Spica. (**29**)<br><br>Syrupus d.	Flowering aerial parts	9: [12]<br><br>11: [9]<br><br>19: [16]<br><br>24: [20]<br><br>29: [23]<br><br>96: [13,19]	Aperitive, digestive<br><br>Anti-inflammatory<br><br>Anti-tubercolosis<br><br>Kidney stones<br><br>Depurative<br><br>Blood depurative<br><br>Laxative<br><br>Airways (non-specified)<br><br>Gastrointestinal disorders (non-specified)<br><br>Reinforces nervous system	Anti-asthma, anti-inflammatory, antioxidant: [184]		

	Artemisie q.p. (**96**)			Rubefacient<br>Thermogenic			
Melissa officinalis L.<br>*Melissa, lemon balm*	Aqua Melisse. (**123**)	Leaves Flowering aerial parts	[66,67,285, 286]	Spasmolytic<br>Tonic	Spasmolytic: [191]		
Mentha aquatica L.<br>*Menta acquatica, water mint*	Oleum Sampsuc. (**101**)	Leaves	[49,285,286]	Anticonvulsant<br>Facilitates birth (Increases the contractions)<br>Paralysis			
Mentha pulegium L.<br>*Puleggio, pennyroyal*	Syrupus d. Artemisie q.p. (**96**)	Aerial parts	[13,19]	Blood depurative<br>Reinforces nervous system	Antimicrobial: [193]<br>Circulatory system: [192]		
Mentha *spp.*<br>*Menta, mint*	Emplastrum crustae panis m. (**78**)	Leaves	[9]	Antibacterial, disinfectant, astringent (wounds)<br>Circulatory system			
Origanum dictamnus L.<br>*Dittamo di creta, dittany of Crete*	Syrupus d. Artemisie q.p. (**96**)	Non specificato	[13,19]	Blood depurative<br>Reinforces nervous system			
Origanum majorana L.<br>*Maggiorana, sweet marjorum*	Syrupus d. Artemisie q.p. (**96**)<br>Oleum Sampsuc. (**101**)	Non specificato Leaves	96: [13,19]<br>101: [49]	Anticonvulsant<br>Blood depurative<br>Facilitates birth (Increases the contractions)<br>Paralysis	Reinforce nervous system (Anti-depressant): [208]	Reinforce nervous system (anti-depressant): Essential oil: involves dopaminergic (D1 and D2), serotonergic (5HT1A, 5-HT2A	Reinforce nervous system (anti-depressant): [208]

				Reinforces nervous system	receptors), and noradrenergic ($\alpha 1$ and $\alpha 2$ adrenoceptors) systems.	
Origanum vulgare L.<br><br>*Origano, oregano*	Electuarium d. Bac. laur. (**54,80**)<br><br>Syrupus d. Artemisie q.p. (**96**)	Leaves	54, 80: [9]<br><br>96: [13,19]	Carminative<br><br>Blood depurative<br><br>Diuretic<br><br>Emmenagogue<br><br>Reinforces nervous system		
Rosmarinus officinalis L.<br><br>*Rosmarino, rosemary*	Unguentum Pectorale (**15**)	Leaves	[13]	Airways	Antimicrobial: [243,244]<br><br>Anti-inflammatory ed Analgesic [243]	Antimicrobial: inhibits mono and polymicrobial biofilms.	Antimicrobial: [244]
Salvia officinalis L.<br><br>*Salvia, sage*	Aqua Pulmon. (**143**)	Leaves	[9]	Circulatory system		
Stachys officinalis (L.) Trevis.<br><br>*Betonica, betony*	Syrupus Betton. (**97**)<br><br>Syrupus d. s. Betton. (**99**)<br><br>Syrupus Betonic. (**116**)<br><br>Aqua Betton. (**132**)	Epigeal parts	97, 99, 116: [9,285,286]<br><br>132: [71]	Anti-depressant, antipsychotic, effects on CNS, mental illness<br><br>Diuretic		
Teucrium scordium L.	Electuarium Diacurcumae (**9**)	Epigeal parts	9: [12]<br><br>48: [32,33]	Antibacterial, Disinfectant (wounds)<br><br>Antipyretic	Gastrointestinal disorders: [272]	Gastrointestinal disorders: reduces oxidative stress in pancreatic islets	Gastrointestinal disorders: [272]

Scordio, water germander	Electuarium Diascord. (**48**)			Diaphoretic<br><br>Airways (non-specified)<br><br>Gastrointestinal disorders (non-specified)<br><br>Thermogenic			
***Thymus* spp**<br><br>*Timo, thyme*	Conserva Hamech (**14**)<br><br>Oleum Sampsuc. (**101**)	Leaves	14: [9]<br><br>101: [49]	Anticonvulsant<br><br>Paralysis<br><br>Skin diseases: scabies (parasiticide), scall, irritations<br><br>Scurvy<br><br>Syphilis (cutaneous symptoms only)<br><br>Stimulates birth (increases the contractions)	Anticonvulsant: [274]<br><br>Skin diseases (danni da radiazione UVB): [275]		
Vitex agnus-castus L.<br>*Agnocasto, chaste tree*	Trochiscus d. Cappar. (**45**)	Seeds	[9]	Anti-inflammatory<br><br>Depurative<br><br>Diuretic<br><br>Emmenagogue	Anti-inflammatory: [280]		
				**Lauraceae**			
Cinnamomum camphora (L.) J. Presl<br><br>*Canfora, camphor*	Unguentum Lapatӱ (**6**)<br><br>Unguentum Citrini (**44**)<br><br>Unguentum	Leaves Scorza	6, 76: [10,11]<br><br>44: [30]	Antifungal (tricofito)<br><br>Parasiticide (parasitosis that causes intermittent fever, i.e., malaria)<br><br>Antipyretic<br><br>Anti-inflammatory	Antifungal: [132]<br><br>Anti-inflammatory: [133]<br><br>Parasiticide: [134]	Anti-inflammatory: Essential oil: reduces TNF-α, IL-1β, and IL-6 levels.	Anti-inflammatory: [133]

	Lapat. (**76**)			Antiviral (herpes)<br><br>Skin blemishes (scars, freckels, Irritations)<br><br>Scurvy			
Cinnamomum verum J.Presl<br><br>*Cannella, cinnamon*	Electuarium Diacurcumae (**9**)<br><br>Syrupus d. Artemisie q.p. (**96**)	Fusto (senza 'sughero' esterno, inteso senza scorza)	9: [12]<br><br>96: [13,19]	Blood depurative<br><br>Airways (non-specified)<br><br>Gastrointestinal disorders (non-specified)<br><br>Reinforce nervous system<br><br>Thermogenic	Airways: [135]<br><br>Gastrointestinal disorders(Anthelmintic): [137]<br><br>Reinforce nervous system: [136]	Airways: cinnamaldehyde: inhibits IL-1β and TNF-*α* production of macrophages stimulated by LPS.	Airways: [135]
Laurus nobilis L.<br><br>*Alloro, laurel*	Electuarium d. Bac. laur. (**54, 80**)	Fruit	[9]	Carminative<br><br>Diuretivo<br><br>Emmenagogue			
				**Leguminosae**			
Acacia senegal (L.) Willd.<br><br>*Gomma arabica, arabic gum*	Diatrium. santal. (**50**)	Gum	[9]	Anti-inflammatory<br><br>Depurative<br><br>Tonic	Anti-inflammatory: [81]	Anti-inflammatory: improves antioxidant levels.	Anti-inflammatory: [81]
Astragalus bustillosii Clos<br><br>*Astragalo, astragalus*	Diatrium. santal. (**50**)	Gum	[9]	Anti-inflammatory<br><br>Depurative<br><br>Tonic			
Galega officinalis L.	Electuarium Diascord. (**48**)	Epigeal parts	[32,33][32, 33]	Antibacterial, Disinfectant (wounds)<br><br>Antipyretic	Antibacterial: [172]		

Galega, goat's-rue				Antiviral<br><br>Respiratory system<br><br>Diaphoretic			
Glycyrrhiza glabra L.<br><br>*Liquirizia, liquorice*	Electuarium Diacurcumae (**9**)<br><br>Diatrium. santal. (**50**)<br><br>Pilulae de Amon. q. (**81**)	Hypogeal parts	9: [12]<br><br>50: [9]<br><br>81: [42]	Antibacterial, disinfetttante (wounds)<br><br>Anti-inflammatory<br><br>Antihistamine<br><br>Depurative<br><br>Diuretic<br><br>Expectorant<br><br>Laxative<br><br>Airways (non-specified)<br><br>Gastrointestinal disorders (non-specified)<br><br>Scabies (Parasiticide)<br><br>Thermogenic<br><br>Tonic	Antibacterial, Anti-inflammatory, Parasiticide: [174]<br><br>Antihistamine: [175]<br><br>Airways (with *Schisandra chinensis* against pulmonary fibrosis): [176]	Anti-inflammatory: inhibits phospholipase A2.<br><br>Antihistamine: Glycyrrhizic acid: suppresses levels of IL-4 (restores immune balance TH1/TH2). Attenuates B cells production of IgE and IgG1.<br><br>Airways: inhibits TGF-β1/Smad2 signaling pathways and overexpresses NOX4	Anti-inflammatory: [174]<br><br>Antihistamine: [175]<br><br>Airways: [176]
Lupinus albus L.<br><br>*Lupino bianco, white lupine*	Trochiscus de Mirra (**51**)	Seeds (senza tegumento esterno)	[9]	Emmenagogue<br><br>Facilitates birth (Increases the contractions)<br><br>Menopausal flushing			
Pterocarpus santalinus L.f.	Diatrium. santal. (**50**)<br><br>Emplastru	Legno (senza scorza)	50, 78: [9]	Antibacterial, Disinfectant, Astringent (wounds)<br><br>Anti-inflammatory	Antibacterial: [233]<br><br>Anti-inflammatory: [232]	Anti-inflammatory: free radicals scavenger	Anti-inflammatory: [232]

Sandalo rosso, red sanders	m crustae panis m. (**78**)			Circulatory system (microcirculation)<br><br>Depurative<br><br>Tonic			
***Trigonella foenum-graecum* L.**<br><br>Fieno greco, fenugreek	Unguentum Dialthee sub. (**61**)	Seeds	61: [36,37]	Anti-inflammatory	Anti-inflammatory: [276]	Anti-inflammatory: Linolenic acid: inhibits LOX and COX.	Anti-inflammatory: [276]
			**Liliaceae**				
Lilium candidum L.<br><br>*Giglio bianco, white lily*	Oleum Lil. alb. q.pl. (**36**)<br><br>Oleum Lil. alb. (**95**)<br><br>Aqua Flor. lil. alb. (**145**)	Hypogeal parts<br>Flowers	36, 95: [28]<br><br>145: [61]	Anti-inflammatory<br><br>Myorelaxant, Spasmolytic	Anti-inflammatory: [185]		
***Lilium* spp.**<br><br>*Giglio, lily*	Flores Lil. Com. (**147**)	Flowers	147: [61]				
			**Linaceae**				
Linum usitatissimum L.<br><br>*Lino, linseed*	Unguentum Dialthee sub. (**61**)	Seeds	61: [37]	Anti-inflammatory	Anti-inflammatory: [186]	Anti-inflammatory: inhibits COX-1 and COX-2	Anti-inflammatory: [186]
			**Loranthaceae**				
Loranthus europaeus Jacq.	Extractus Vissi. querc. (**60**)	Fruit	[35,285,286]	Anti-inflammatory<br><br>Sore throat			

Vischio, European loranth							
				**Malvaceae**			
***Althaea officinalis* L.**<br><br>*Altea, marsh mallow*	Unguentum Pectorale (**15**)<br>Unguentum Dialthee sub. (**61**)<br>Syrupus Althee. fernet. (**98**)	Hypogeal parts	15: [13]<br><br>61: [37]<br><br>98: [9]	Anti-inflammatory<br><br>Expectorant<br><br>Mucolytic	Anti-inflammatory: [99]<br><br>Expectorant: [100]	Anti-inflammatory: inhibits LPS-induced production of TNF-α and IL6 in macrophages.	Anti-inflammatory: [99]
				**Myristicaceae**			
***Myristica fragrans* Houtt.**<br><br>*Noce moscata, macis, nutmeg, mace*	Unguentum Pectorale (**15**)<br><br>Oleum Nucis. myrist. (**18**)<br><br>Emplastrum crustae panis m. (**78**)<br><br>Electuarium bened. lax. (**19**)	Fruit, mesocarpo Seeds	15: [13]<br><br>18: [15,285,286]<br><br>19: [16]<br><br>78: [9]	Analgesic<br><br>Antibacterial, disinfectant, astringent (wounds)<br><br>Anticonvulsant<br><br>Antinfiammtorio<br><br>Kidney stones, gravel<br><br>Diuretic<br><br>Circulatory system<br><br>Facilitates birth (Increases the contractions)<br><br>Laxative<br><br>Respiratory system infections, colds<br><br>Stomach-ache (external use)	Analgesic, Anti-inflammatory: [194,197]<br><br>Antibacterial: [194–196]<br><br>Anticonvulsant: [194]<br><br>Antioxidant: [195,196]<br><br>Stomach-ache: [196]	Analgesic: amphetamine-like activity<br><br>Anticonvulsant: decreases dopaminergic transmission<br><br>Stomach-ache: antibacterial activity against *Helicobacter pylori*.	Analgesic, anticonvulsant: [194]<br><br>Stomach-ache [196]

				Tonic			
				**Myrtaceae**			
Myrtus communis L.<br><br>*Mirto, myrtle*	Trochiscus d. Myrtha. (**13**)<br><br>Syrupus de Myrtio (**32**) (**Ingrediente di: Syrupus Myrtinus compositus**)<br><br>Oleum Sampsuc. (**101**)	Fruit	32: [9]<br><br>101: [21]	Anticonvulsant<br><br>Astringent, wound healing, hemorrhages<br><br>Digestive<br><br>Facilitates birth (Increases the contractions)<br><br>Paralysis	Antioxidant: [199,200]<br><br>Antiulcer, neuroprotettivo: [198]	Antiulcer: reduces gastric juice volume and total acidity. Increases gastric pH and gastric wall mucus content<br><br>Neuroprotector: protective antioxidant activity.	Antiulcer, neuroprotector: [198]
				**Oleaceae**			
Ligustrum vulgare L.<br><br>*Ligustro, common privet*	Syrupus d. Artemisie q.p. (**96**)	Seeds	[13,19]	Blood depurative<br><br>Reinforces nervous system			
Olea europaea L.<br><br>*Olivo, olio, olive, oil*	Oil, ingredient of several preparations, such as:<br><br>Oleum Sampsuc. (**101**)<br><br>Oleum de. Rutha. (**102**)<br><br>Oleum Hyperic. q.pl. (**104**)	Fruit, oil	101: [49]<br><br>102: [50]<br><br>104: [52]<br><br>112: [9]	Analgesic<br><br>Anaesthetic (local)<br><br>Antibacterial, Disinfectant (wounds)<br><br>Anticonvulsant<br><br>Anti-inflammatory<br><br>Facilitates birth (Increases the contractions)<br><br>Otitis<br><br>Paralysis	Antibacterial: [206,207] -olive mill wastewater – extravirgin olive oil by-product [205]<br><br>Anti-inflammatory: [111,205,207]	Anti-inflammatory: Phenolic compounds: Inhibit COX-1 and COX-2. Suppress PG-E2. Inhibits NF-kB translocation in human monocytes. Protect DNA from oxidative damage. Reduce NF-kB activation, improve NF-kB inhibitors.	Anti-inflammatory: [207]

	Oleum de. caparb. s. (**112**)					Inhibit COX-2, iNOS, and LOX.	
**Papaveraceae**							
Fumaria officinalis L.<br>*Fumaria, drug fumitory*	Syrupus rosatus solutus cum fumaria (**28**)<br>Syrupus rosatum cum fumaria (**105**)<br>Aqua Fumar. (**146**)	Aerial parts	28, 105, 146: [8]<br>146: [47,286]	Anti-inflammatory<br>Kidney stones	Anti-inflammatory: [171]<br>Diuretic: [170]	Anti-inflammatory: possibly reduces TNF-α and IL-6. Improves anti-inflammatory substances (IL-10) and oxidative stress.	[171]
Papaver somniferum L.<br>*Papavero da oppio, opium poppy*	Electuarium Diacurcumae (**9**)<br>Opiatus poter. (**55**)<br>Pilulae de Cinoglo. (**69**)	Unripe fruit<br>Gum<br>Seeds	9: [12]<br>55: [34]<br>69: [9]	Analgesic<br>Astringent for gums<br>Expectorant<br>Sedative-hypnotic<br>Narcotic<br>Gastrointestinal disorders (non-specified)<br>Reinforces nervous system<br>Thermogenic	Airways: [209]<br>Analgesic: [210,211,213,297]<br>Antidiarrhoeal, Sedative: [210]<br>Eccitante: [211]<br>Neuroprotezione: [212]	Airways: Opioids: reduce neuronal rhythmic activity, inducing respiratory depression.<br>Analgesic: opioids interaction with μ receptors.<br>Neuroprotector: upregulating BDNF-TrkB signaling and thus, reducing TNF-α neurotoxicity.<br>Sedative: opioids anticholinergic activity.	Airways: [209]<br>Analgesic: [297]<br>Neuroprotector: [212]<br>Sedative: [210]
**Pedaliaceae**							

Sesamum indicum L.<br><br>*Sesamo, sesame*	Oleum Spica. (**29**)	Seeds, oil	[23]	(Sesame oil as a carrier in the preparation)<br><br>Astringent (cutaneous)<br><br>Rubefacient			
				**Pinaceae**			
Larix spp.<br><br>or<br><br>***Pinus* spp.**<br><br>or<br><br>***Picea* spp.**<br><br>*Trementina, colofonia, turpentine, colophony*	Unguentum Dialthee sub. (**61**)	Resin	[37]	Airways, expectorant<br><br>Antibacterial, Disinfectant (wounds)<br><br>Anti-inflammatory<br><br>Depurative<br><br>Diuretic<br><br>Laxative<br><br>Mucolytic<br><br>Scabies (Parasiticide)	Antibacterial, antifungal: [183] -colofonia [181,182]<br><br>Airways, anti-inflammatory, antioxidant, depurative, diuretic: [181]	Airways: smooth muscle relaxant, cough preventing.	Airways: [181]
				**Piperaceae**			
Piper longum L.<br><br>*Pepe longo, Indian long pepper*	Electuarium bened. lax. (**19**)<br><br>Electuarium d. Bac. laur. (**54,80**)	Fruit	19: [16]<br><br>54,80: [9]	Carminative<br><br>Diuretic<br><br>Emmenagogue<br><br>Laxative	Anti-inflammatory, Antioxidant: [218]		
Piper nigrum L.<br><br>*Pepe nero, black pepper*	Unguentum artanita (**24**)<br><br>Electuarium d. Bac. laur. (**54,80**)	Fruit	24: [20]<br>54, 80: [9]	Carminative<br><br>Stomach depurative<br><br>Diuretic<br><br>Emmenagogue<br><br>Laxative<br><br>Vermifuge	Anti-inflammatory: [219,221]<br><br>Antioxidant: [219,220]<br><br>Parasiticide: [221]<br><br>Digestive: [219]		

**Plantaginaceae**							
***Veronica* spp.** *Veronica*	Opiatus poter. (**55**) Aqua Pulmon. (**143**)	Non specificato Buds	55: [34] 143: [9]	Analgesic Circulatory system Narcotic	Anti-inflammatory: [278]		
**Poaceae**							
Hordeum vulgare L. *Orzo, barley*	Oleum Sup. hord. (**26**)	Seeds (oil)	[21]	Intestinal motility	Antidiarrhoeal: [177,178] - Epigeal parts, whole seeds) Constipation: [177] - Whole seeds	Antidiarrhoeal: modifies gut microbiota, promotes growth of *Prevotella* and *Anaerovibrio* genuses Constipation: increases fecal mass	Antidiarrhoeal: [177] Constipation: [178]
Triticum aestivum L. subsp. ***aestivum*** *Grano, wheat*	Emplastrum crustae panis m. (**78**)	Fruit	[9]	Antibacterial, Disinfectant, Astringent (wounds) Circulatory system	Antioxidant, cardioprotctor, circulatory system: (Sharma et al., 2014 - Leaves)	Cardioprotector: increases the function of the queen, reduces total cholesterol, LDL. Increases HDL.	Cardioprotector: [277]
**Polygonaceae**							
Rheum officinale L. *Rabarbaro, rhubarb*	Electuarium Diacurcumae (**9**) Syrupus Cichor. Com. Reub. Gul. -n. (**94, 107**)	Hypogeal parts Epigeal parts	9: [12] 94, 107: [47,48]	Airways (non-specified) Gastrointestinal disorders (non-specified) Thermogenic	Airways, anti-inflammatory, gastrointestinal disorders, thermogenic: [237] Antioxidant: [236]	Airways: decreases capillary permeability to albumin, myeloperoxidase activity, NO concentration, phospholipase-A2 activity, and TNF levels.	Airways: [237]
Rumex conglomeratus Murray *Lapazio acuto,*	Unguentum Lapatӱ (**6**)	Leaves Epigeal parts	6,76: [10,11]	Antifungal Anti-inflammatory Antiviral (herpes)	Antibacterial: [248] Antioxidant: [247]		

clustered dock	Unguentum Lapat. (**76**)						
			**Polypodiaceae**				
Polypodium vulgare L.<br>*Polipodio, polypody*	Conserva Hamech. (**14**)<br>Unguentum artanita (**24**)	Hypogeal parts<br>Leaves	14: [9]<br>24: [20]	Stomach depurative<br>Laxative<br>Skin diseases: Scabies (parasiticide), scall, itch, and irritations<br>Scurvy<br>Syphilis (cutaneous symptoms only)<br>Vermifuge	Analgesic, Antibacterial, Antiviral, Digestive, laxative, Scurvy: [224]		
			**Portulacaceae**				
Portulaca oleracea L.<br>*Portulaca, common purslane*	Diatrium. santal. (**50**)<br>Syrupus d. Artemisie q.p. (**96**)	Hypogeal parts<br>Epigeal parts	50: [9]<br>96: [13,19]	Anti-inflammatory<br>Blood depurative/Lymphatic system<br>Effects on Nervous System<br>Tonic	Anti-hypoxia, anti-inflammatory, antioxidant, hepatoprotector, neuroprotector: [225]	Anti-inflammatory: inhibits TNF-α levels and NF-κB translocation.	Anti-inflammatory: [225]
			**Primulaceae**				
Cyclamen hederifolium Aiton<br>*Ciclamino, cyclamen*	Unguentum Artanita (**24**)	Epigeal parts	[20]	Antibacterial (Tuberculosis, ointment)<br>Anti-inflammatory<br>Stomach depurative<br>Laxative<br>Vermifuge	Anti-inflammatory: [151]		
			**Pteridaceae**				

***Adiantum capillus-veneris* L.** *Capelvenere comune, southern maidenhair fern*	Syrupus cap. ven. (**103**)	Epigeal parts	[51,285,286]	Anti-inflammatory<br>Antipyretic<br>Antiviral (Herpes)	Antimicrobial, Antiviral: [87]<br>Anti-inflammatory: [88–90]<br>Antipyretic: [88] (polyherbaceous formulation)	Anti-inflammatory: Flavonoids, sterols, terpenoids: possibly decreases hystamine, 5HT, prostaglandines, bradykinins. Suppresses NF-κB.	Anti-inflammatory: [89,90]
				**Ranunculaceae**			
***Nigella damascena* L.** *Nigella damascena, love-in-a-mist*	Trochiscus d. Cappar. (**45**)	Seeds	[9]	Anti-inflammatory<br>Kidney stones<br>Diuretic<br>Emmenagogue	Antioxidant, Diuretic: [203]	Diuretic: increases diuresis volume and Na^+ and K^+ excretion.	Diuretic: [203]
***Nigella sativa* L.** *Nigella, black cumin*	Electuarium d. Bac. laur. (**54,80**)	Seeds	54, 80: [9]	Carminative<br>Diuretic<br>Emmenagogue	Anti-inflammatory, Emmenagogue, estrogenico: [204]<br>Antioxidant: [203,204]<br>Diuretic: [203]		
				**Rosaceae**			
***Agrimonia eupatoria* L.** *Agrimonia, agrimony*	Trochiscus d. Cappar. (**45**)<br>Aqua Pulmon. (**143**)	Hypogeal parts<br>Buds	45, 143: [9]	Anti-inflammatory<br>Diuretic<br>Circulatory system<br>Emmenagogue	Anti-inflammatory, Antioxidant: [93] - Aerial parts		
***Malus domestica* Borkh.** *Melo, Mela renetta, apple*	Syrupus d. Pomid.s. (**30**)<br>Pulvis Flor. Malu. (**113**)	Flowers<br>Fruit	30: [9]<br>113: [14]	Antidiarrhoeal<br>Expectorant	Antidiarrhoeal: [188] (unripe apples)<br>Anti-inflammatory: [187]<br>Airways:	Antidiarrhoeal: Polyphenols: inhibit ADP ribosylation of agmatine (induced by cholera toxin). Inhibit	Antidiarrhoeal: [188]<br>Anti-inflammatory: [187]

					[189]	enzymatic activity of cholera toxin.<br><br>Anti-inflammatory: inhibit NF-κB.	
Potentilla erecta (L.) Raeusch.<br><br>*Tormentilla, tormentil*	Electuarium Diascord. (**48**)	Hypogeal parts	48: [32,33]	Antibacterial, Disinfectant (wounds)<br><br>Antipyretic<br><br>Antiviral<br><br>Diaphoretic	Antimicrobial: [226]		
Prunus domestica L.<br><br>Prugna, plum	Conserva Hamech. (**14**)	Scorza, balsamo	[9]	Antihistamine<br><br>Skin diseases: Scabies (parasiticide), scall, itch, and irritations<br><br>Scurvy<br><br>Syphilis (cutaneous symptoms only)	Antihistamine: [227]		
Prunus dulcis (Miller) D.A. Webb<br><br>*Mandorlo dolce, sweet almond*	Unguentum Pectorale (**15**)<br><br>Unguentum artanita (**24**)<br><br>Syrupus d. Pomis. s. (**30**)<br><br>Trochiscus d. Cappar. (**45**)<br><br>Electuarium d.	<u>Seeds, oil</u>	15: [13]<br><br>24: [20]<br><br>30, 45, 54, 80: [9]<br><br>106: [53,285,286]	Antidiarrhoeal<br><br>Anti-inflammatory<br><br>Carminative<br><br>Stomach depurative<br><br>Diuretic<br><br>Emmenagogue<br><br>Emollient<br><br>Expectorant<br><br>Laxative<br><br>Tuberculosis (ointment)<br><br>Vermifuge	Anti-inflammatory: [228] - <u>seeds skins</u><br><br>Emollient: [229] - <u>Leaves</u> [230]	Anti-inflammatory: inhibits IL-17, upregulating IFN-α and IL-4.	Anti-inflammatory: [228]

	Bac. laur. (**54,80**)<br><br>Oleum Amigda. dul. (**106**)						
Prunus persica (L.) Batsch<br><br>*Pesca, peach*	Syrupus Flor. Pers. (**110**)<br><br>Aqua Gland. persic. (**142**)	Flowers Seeds	110: [14,55,285, 286]<br><br>142: [10,11]	Carminative<br><br>Choleretic<br><br>Laxative<br><br>Myorelaxant<br><br>Vermifuge	Laxative: [231]	Laxative: calcium channels blockage.	Laxative: [231]
***Rosa* spp.**<br><br>*Rosa, rose*	Unguentum Lapatÿ (**6**)<br><br>Unguentum Rosati (**10**)<br><br>Trochiscus Absÿnthi (**11**)<br><br>Conserva Hamech. (**14**)<br><br>Electuarium bened. lax. (**19**)<br><br>Syrupus rosatum cum fumaria (**28**)	Flowers	6, 10, 76, 93, 100, 115: [10,11]<br><br>11, 14, 50, 84, 143: [9]<br><br>19: [16]<br><br>28, 105: [8]<br><br>35: [27]<br><br>86: [44]<br><br>119: [58]	Antibacterial, Disinfectant<br><br>Antifungal<br><br>Anti-inflammatory<br><br>Antipyretic<br><br>Antiviral (herpes)<br><br>Aperitive, digestive<br><br>Wound healing<br><br>Choleretic<br><br>Liver depurative<br><br>Stomach depurative<br><br>Diaphoretic<br><br>Diuretic<br><br>Emmenagogue<br><br>Expectorant<br><br>Jaundice	Antibacterial: [238,239,242]<br><br>Anti-inflammatory: [241,242]<br><br>Laxative: [240]<br><br>Antiviral: [242]	Anti-inflammatory: free radicals scavenger, lowers superoxide anion.	Anti-inflammatory: [242]

Syrupus heder. terres. (**35**)<br><br>Diatrium. santal. (**50**)<br><br>Unguentum Lapat. (**76**)<br><br>Conserva Rosar. (**84**)<br><br>Pilulae Masticin. (**86**)<br><br>Mel. Ros. sol. com. (**93**)<br><br>Syrupus roxato (**100**)<br><br>Syrupus rosatum cum fumaria (**105**)<br><br>Syrupus ex. Trib. infus. (**115**)<br><br>Mel. Ros. simp. (**119**)	Laxative<br><br>Stomach-ache<br><br>Skin diseases: scabies (parasiticide), scall, itch, and irritation<br><br>Scurvy<br><br>Syphilis (cutaneous symptoms only)<br><br>Tonic

	Aqua Pulmon. (**143**)						
Rubus ulmifolius Schott<br>*Mora, elm-leaf blackberry*	Syrupus Diamor. (**111**)	Fruit	[13]	Anti-inflammatory	Anti-inflammatory: [246]	Anti-inflammatory: inhibits COX-2 gene expression.	Anti-inflammatory: [246]
Sanguisorba officinalis L.<br>*Sanguisorba, great burnet*	Aqua Pulmon. (**143**)	Buds	[9]	Circulatory system	Circulatory system: [262]	Circulatory system: Saponins: improve hematopoiesis by promoting survival through FAK and Erk1/2 activation and modulating cytokine production in the bone marrow.	Circulatory system: [262]
				**Rubiaceae**			
Rubia tinctorum L.<br>*Robbia comune, madder*	Electuarium Diacurcumae (**9**)<br>Trochiscus de Mirra (**51**)	Hypogeal parts<br>Flowers	9: [12]<br>51: [9]	Emmenagogue<br>Facilitates birth (increases the contractions)<br>Airways (non-specified)<br>Gastrointestinal disorders (non-specified)<br>Menopausal flushing<br>Thermogenic	Gastrointestinal disorders (Anti-diarrhoeal): [245]	Gastrointestinal disorders: possibly antispasmodic effect, with reduction of intestinal contractions, allowing more time for water absorption.	Gastrointestinal disorders: [245]
				**Rutaceae**			
Citrus limon (L.) Osbeck	Syrupus de s. Citri. (**108**)	Fruit, epicarpo	108: [44]				

Limone, lemon	Syrupus de Suc. cit. (**149**)						
Citrus medica L.<br><br>*Cedro, citron*	Unguentum Citrini. (**44**)<br><br>Aqua. Tot. citri. (**135**)	Fruit	44: [30,285,286]<br><br>135: [77,285,286]	Antibacterial<br><br>Skin blemishes (scars, freckels, irritations)<br><br>Spasmolytic	Antibacterial: [131]		
Ruta graveolens L.<br><br>*Ruta, strong smelling rue*	Trochiscus d. Cappar. (**45**)<br><br>Electuarium Diascord. (**48**)<br><br>Electuarium d. Bac. laur. (**54, 80**)<br><br>Syrupus d. Artemisie q.p. (**96**)<br><br>Oleum de. Rutha. (**102**)<br><br>Aqua Ruth. orten. (**124**)	Leaves	45, 54,80: [9]<br><br>48: [32,33]<br><br>96: [13,19]<br><br>102: [50,285,286]<br><br>124: [68,285]	Analgesic<br><br>Antibacterial, Disinfectant (wounds)<br><br>Anti-inflammatory<br><br>Antipyretic<br><br>Aperitive, digestive<br><br>Carminative<br><br>Cephalea<br><br>Blood depurative<br><br>Diaphoretic<br><br>Diuretic<br><br>Emmenagogue<br><br>Reinforces nervous system<br><br>Spasmolytic	Antibacterial: [250]<br><br>Antiinfiammatorio: [249]<br><br>Antipyretic: [249]		
				**Santalaceae**			
Santalum album L.<br><br>*Sandalo, sandalwood*	Diatrium. santal. (**50**)<br><br>Emplastrum	Legno, senza scorza	50: [285,286]<br><br>50, 78: [9]	Antibacterial, disinfectant, astringent (wounds)<br><br>Anti-inflammatory	Antibacterial: [263]<br><br>Anti-inflammatory (neuro-inflammation): [264]	Anti-inflammatory (neuro-inflammation): increases expression of IFN-β and IFN-α.	Anti-inflammatory: [264]

	crustae panis m. (**78**)			Circulatory system<br>Tonic		Reduces expression of IL-6, CXCL8, CCL2, and IP-10. Causes over-expression of TLR2 and TLR4.
			**Saxifragaceae**			
Saxifraga *spp*<br>*Sassifraga, saxifrage*	Electuarium bened. lax. (**19**)	Seeds	[16]	Laxative		
			**Solanaceae**			
Hyoscyamus niger L.<br>*Giusquiamo, henbane*	Pilulae de Cinoglo. (**69**)	Seeds	[9]	Astringent for gums<br>Expectorant<br>Sedative-hypnotic		
			**Tamaricaceae**			
Tamarix gallica L.<br>*Tamerice comune, French tamarisk*	Unguentum artanita (**24**)	Fruit	[20]	Anti-inflammatory<br>Tuberculosis (ointment)		
			**Thymelaeaceae**			
Daphne mezereum L.<br>*Dafne mezereo, February daphne*	Unguentum artanita (**24**)	Scorza	[20]	Stomach depurative<br>Laxative<br>Vermifuge		
			**Violaceae**			
Viola tricolor L. subsp. ***tricolor***<br>*or*	Unguentum Lapatÿ (**6**)<br>Conserva	Flowers	6, 76: [10,11]<br>14, 50, 69: [9]	Antifungal<br>Anti-inflammatory<br>Antiviral (herpes)	Anti-inflammatory: [279]	

***Viola* spp.**<br><br>*Viola, violet*	Hamech (**14**)<br><br>Diatrium. santal. (**50**)<br><br>Pilulae de Cinoglo. (**69**)<br><br>Unguentum Lapat. (**76**)			Astringent for gums<br><br>Expectorant<br><br>Sedative-hypnotic<br><br>Skin diseases: scabies (parasiticide), scall, Itch, and irritations<br><br>Scurvy<br><br>Syphilis (cutaneous symptoms only)<br><br>Tonic	
**Vitaceae**					
***Vitis vinifera* L.**<br><br>*Vino, aceto, wine, vinegar*	Il vino o l'aceto sono ingredienti di diversi preparati tra cui:<br><br>Unguentum artanita (**24**)<br><br>Oxymel scyll. (**27**)<br><br>Oleum Spica. (**29**)<br><br>Oxymel Simpl. (**117**)<br><br>Aqua Gland.	Fruit, vino o aceto	24: [20]<br><br>27: [22]<br><br>29: [23]<br><br>117: [56,57]<br><br>142: [10,11]	Anti-inflammatory<br><br>Carminative<br><br>Stomach depurative<br><br>Emetic<br><br>Expectorant, mucolytic<br><br>Laxative<br><br>Stomach-ache<br><br>Tuberculosis (ointment)<br><br>Vermifuge	Anti-inflammatory: [281]

	persic. (**142**)						
				**Xanthorrhoeaceae**			
Aloe perryi Baker<br><br>*Aloe socotrina, Socotrine aloe*	Pilulae Masticin. (**86**)	Leaves, juice	86: [44]	Laxative			
***Aloe* spp.**<br><br>*Aloe, aloe*	Unguentum artanita (**24**)<br><br>Pilulae Aloe. lota. (**62**)<br><br>Pilulae de Amon. q. (**81**)	Leaves, juice e gel	24: [20]<br><br>62: [10,11,19]<br><br>81: [42]	Antibacterial, disinfectant (wounds)<br><br>Depurative<br><br>Emmenagogue<br><br>Expectorant, Respiratory tract<br><br>Laxative<br><br>Scabies (Parasiticide)<br><br>Stomach depurative<br><br>Vermifuge	Antibacterial: [96]<br><br>Laxative (Emodin): [98]<br><br>Respiratory tract prophylaxis: [94]<br><br>Vermifuge (Anthelmintic): [97]<br><br>Wound healing: [95]	Laxative: Emodin: increases expression of aquaporin 3.	Laxative: [98]
				**Zingiberaceae**			
Alpinia galanga (L.) Willd.<br><br>*Galanga, Siamese ginger*	Electuarium bened. lax. (**19**)	Hypogeal parts	[16]	Laxative			
Curcuma longa L.<br><br>*Curcuma, turmeric*	Electuarium Diacurcumae (**9**)	Hypogeal parts Leaves	[12]	Airways (non-specified)<br><br>Gastrointestinal disorders (non-specified)<br><br>Myorelaxant<br><br>Thermogenic	Airways: [148,149]<br><br>Gastrointestinal disorders: [149]	Airways (asthma) Curcumin: regulates transcription factors as NF-kB, cytokines (IL6 and TNF-α), adhesion molecules as ICAM-1, and	Airways (asthma): [148]

						enzymes (MMPs) Inhibiting NF-kB activity reduces AHR and inflammatory cell airway infiltration and determines IgE levels attenuation in BALF	
Elettaria cardamomum (L.) Maton<br><br>*Cardamomo, green cardamom*	Electuarium bened. lax. (**19**)	Seeds	[16]	Laxative			
Zingiber officinale Roscoe<br><br>*Zenzero, ginger*	Trochiscus de Agarici (**17**)<br><br>Electuarium bened. lax. (**19**)<br><br>Unguentum artanita (**24**)	Hypogeal parts	17: [10,11,14]<br><br>19: [16]<br><br>24: [20]	Gastric acidity<br><br>Stomach depurative<br><br>Expectorant<br><br>Laxative<br><br>Stomach-ache<br><br>Vermifuge	Gastric acidity: [284]<br><br>Stomach depurative and stomach-ache (gastroprotective effect): [283]<br><br>Vermifuge/Anthelmintic: [282]	Gastric acidity: Phenolic compounds: antioxidant activity, inhibits gastric acid, K^+-ATPase, and *Helicobacter pylori* growth.	Gastric acidity: [284]

Key: Green: the highlighted article cites polar extracts and preparations (water, EtOH, MeOH); Underlined: the underlined article cites plant parts different from the ones in the historical source; Red: the species showed in scientific literature an activity opposite to that in the historical source.
